# Deep Volumetric Super‐Resolution Imaging in Thick Biological Specimens With Sparse Scanning SIM

**DOI:** 10.1002/advs.74516

**Published:** 2026-02-23

**Authors:** Sha An, Xuhong Guo, Zhongxia Cai, Yunze Lei, Kai Wen, Hongfei Suo, Xiaoyu Kong, Gerd Ulrich Nienhaus, Peng Gao

**Affiliations:** ^1^ School of Physics Xidian University Xi'an China; ^2^ Key Laboratory of Optoelectronic Perception of Complex Environment Ministry of Education Xi'an China; ^3^ Engineering Research Center of Information Nanomaterials Universities of Shaanxi Province Xi'an China; ^4^ Institute of Applied Physics Karlsruhe Institute of Technology Karlsruhe Germany; ^5^ Institute of Nanotechnology Karlsruhe Institute of Technology Eggenstein‐Leopoldshafen Germany; ^6^ Institute of Biological and Chemical Systems Karlsruhe Institute of Technology Eggenstein‐Leopoldshafen Germany; ^7^ Department of Physics University of Illinois at Urbana‐Champaign Urbana Illinois USA

**Keywords:** deep super‐resolution imaging, resonant scanning, structured illumination microscopy

## Abstract

High‐resolution fluorescence microscopy deep inside thick, live specimens remains a major challenge due to scattering and limited penetration depth. Structured illumination microscopy (SIM) is widely used for fast super‐resolution imaging, yet its sensitivity to scattering strongly restricts volumetric applications. Here we present sparse scanning SIM (SS‐SIM), a variant for deep imaging that integrates rapid laser beam scanning with pixel‐addressed intensity modulation and sCMOS detection. SS‐SIM operates with either one‐ or two‐photon excitation and achieves a spatial resolution of ∼150 nm, and maintains a 1.6/1.7‐fold (lateral/axial) resolution enhancement over wide‐field microscopy throughout the available imaging depth limited by the working distances of our water‐immersion objectives (300/600 µm), enabling volumetric visualization of dense biological samples. By combining simplicity, robustness, and super‐resolution performance, SS‐SIM expands the applicability of SIM to thick tissues, organoids, and small organisms, providing a practical route to high‐contrast volumetric imaging in complex biological environments.

## Graphical abstract

Sparse scanning structured illumination microscopy (SS‐SIM) extends the axial depth in super‐resolution fluorescence imaging by combining rapid laser scanning, pixel‐addressed intensity modulation, and sCMOS camera detection. SS‐SIM yields 1.6× lateral and 1.7× axial resolution gains over wide‐field microscopy and enables imaging through 300–600 µm depths, supporting practical volumetric imaging of thick samples.

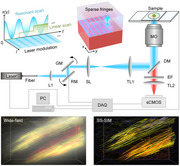

## 1 Introduction

Optical microscopy has long been an indispensable tool for the exploration of biological structure, dynamics, and function [1]. Conventional far‐field imaging, however, is fundamentally constrained by the Abbe diffraction limit [2] restricting the spatial resolution to ∼200–300 nm laterally and ∼500–700 nm axially. Overcoming this barrier, a variety of super‐resolution fluorescence microscopy techniques have been developed in recent years [3, 4, 5] including stimulated emission depletion (STED) microscopy [6, 7], single‐molecule localization microscopy (SMLM) [8, 9] and structured illumination microscopy (SIM) [10, 11, 12].

Among these methods, SIM has gained particular prominence because it enables imaging of large fields of view (FOV) with moderate light intensity and thus low phototoxicity. Unlike other super‐resolution modalities, SIM is compatible with all types of fluorophores. Its axial sectioning and super‐resolution capabilities are rooted in the spatial modulation of the excitation light. In classical SIM, samples are exposed to multiple, spatially periodic patterns of light formed by interference of coherent light beams, from which a single image is reconstructed by computation [13, 14]. In the reconstruction process, high spatial frequency image components are shifted into the diffraction‐limited passband of the microscope by the moiré effect. When exciting fluorophores in the sample with sinusoidal illumination line patterns, SIM provides up to twofold enhanced resolution over conventional widefield imaging. Even higher resolution enhancement is feasible with non‐sinusoidal patterns, e.g., by saturating the emission of fluorophores under intense illumination [15, 16]. All these beneficial properties and its comparatively fast imaging speed render SIM attractive for live imaging applications.

Recent years have seen an increasing demand for fast 3D super‐resolution imaging of thick biological tissues, organoids, and small model organisms. Classical SIM, however, encounters major limitations in this context: scattering and aberrations in thick specimens degrade the intensity modulation contrast essential for accurate image reconstruction, restricting the achievable imaging depth to about 20 µm [17]. Scanning SIM was proposed to enhance the imaging depth [12, 18, 19]. Yet, the linear scan of focus in scanning SIM leads to a long acquisition time (≥10 s) for a single SR image. Meanwhile, the SNR and imaging depth of scanning SIM are limited by the low contrast of the dense fringes (period ∼200 nm) used, which further decreases with the imaging depth.

Here, we introduce sparse scanning structured illumination microscopy (SS‐SIM) for 3D imaging of thick biological specimens at unprecedented depth. The system builds on a standard point‐scanning microscope equipped with a fast resonant scanner and synchronized modulation of the excitation laser intensity, enabling arbitrary 2D excitation patterns to be generated within the integration time of a single sCMOS camera frame. To enable rapid image acquisition, we demonstrate SS‐SIM using horizontal and vertical fringe patterns with either one‐photon (1PE) or two‐photon (2PE) excitation. Unlike conventional SIM, which minimizes the fringe period through wave interference, our approach employs excitation patterns with sharp fringes separated by 2.4 µm, ensuring that scattering from adjacent lines has only a minor effect on modulation contrast. As a result, fringe contrast is preserved over extended axial ranges, permitting high‐quality SS‐SIM imaging throughout the full working distances of our water‐immersion objectives (300/600 µm). The sparse periodic excitation patterns incorporate multiple harmonics of the fundamental spatial frequency, enabling reconstruction of super‐resolved images with ∼1.6‐fold resolution enhancement beyond the Abbe limit. Practically, twelve phase‐shifted patterns are acquired along each lateral dimension within 0.65 s, from which SIM images are reconstructed in a manner analogous to non‐linear SIM. Collectively, these results establish SS‐SIM as a powerful extension of SIM, bridging high‐resolution imaging with the requirements of deep, live‐cell, and tissue investigations.

## 2 Results

### 2.1 Overview of the SS‐SIM Microscope and Structured Illumination

**FIGURE 1:**
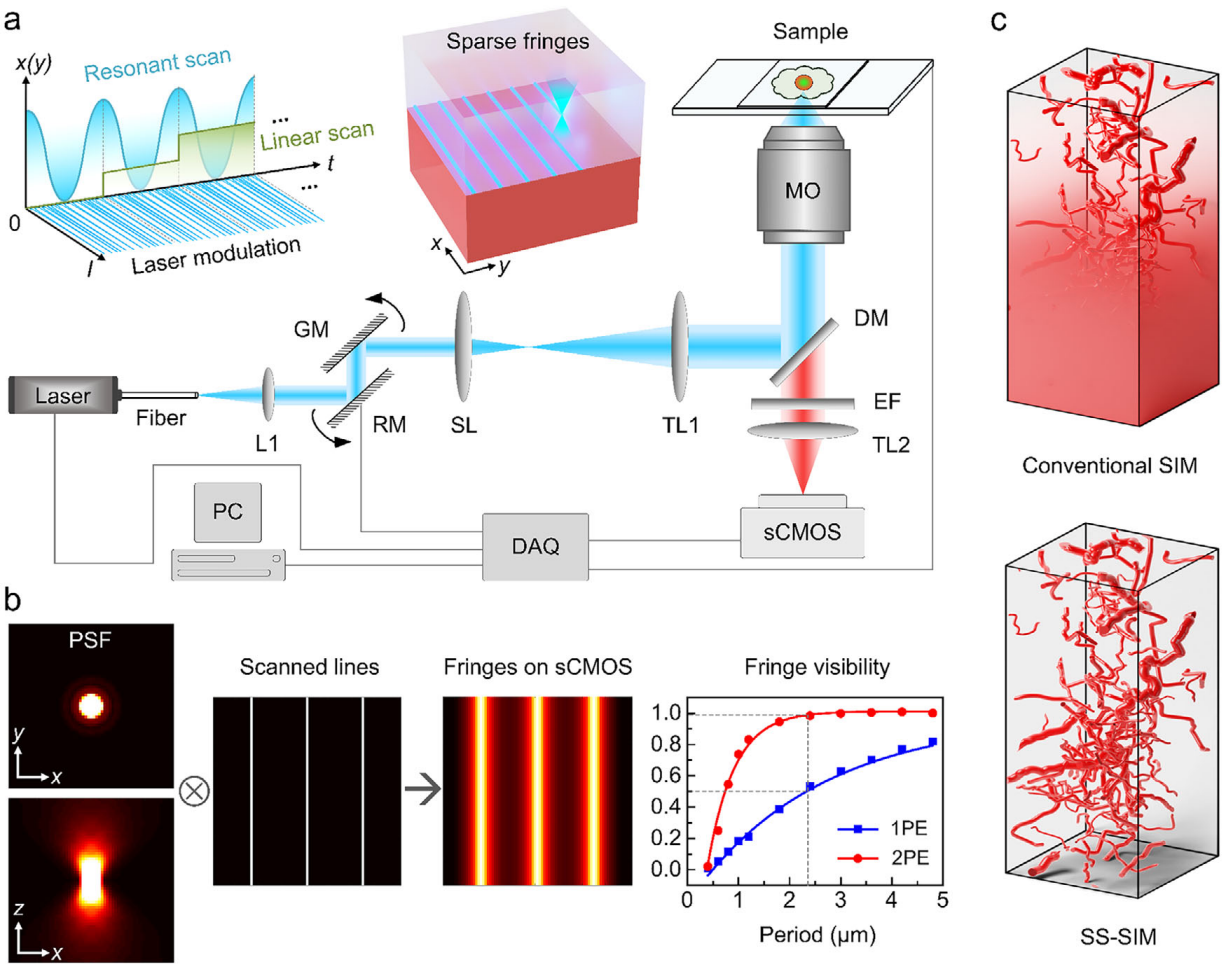
Overview of the SS‐SIM technique. (a) Schematic depiction of the SS‐SIM system. In this fast point‐scanning device, a laser beam (1PE: 640 nm, 2PE: 920 nm) is fiber‐coupled via lens L1 into a beam scanner, which deflects the beam along the *x* dimension with a fast resonant mirror (RM) oscillating at 12 kHz, and along the *y* dimension with a regular, slow‐scanning galvo mirror (GM) performing linear motion. After beam shaping via the scan lens (SL) and a tube lens 1 (TL1), the beam is deflected by a dichroic mirror (DM) into the microscope objective (MO), forming a sharp focus in the sample. The emitted fluorescence is passed through the emission filter (EF) and TL2, and collected on a camera (sCMOS). The setup is controlled by a personal computer (PC) with a data acquisition card (DAQ), enabling proper synchronization of the laser power, the fast *x/y* beam scanner, the stage scanner for *z*‐scanning, and image acquisition by the camera. The delicate interplay between the beam scanner and the laser modulation (upper left) allows for fast generation of diverse spatial excitation patterns (upper middle). (b) The resulting intensity pattern is given by the convolution (⊗) of the 3D point spread function (PSF (left)) of the system with the scanned and modulated laser beam (here lines). On the right, the fringe contrast, or visibility, *V* = (*I*_max_ – *I*_min_)/(*I*_max_ + *I*_min_), of our system is plotted against the fringe period, showing that we require periods of at least 2.4 µm to achieve *V* ≥0.5 for 1PE and *V* ≥0.98 for 2PE. (c) Schematic of 3D image stacks of thick samples taken with conventional SIM and SS‐SIM highlights the enhanced deep imaging capability of SS‐SIM.

Our SS‐SIM apparatus (Figure 1a) is based on a home‐built fluorescence microscope, featuring water immersion objectives compatible with live imaging, pulsed lasers (for 1PE and 2PE), and wide‐field sCMOS camera detection. Details are described in Methods. The laser focus is scanned by a 12 kHz resonant scanning mirror (RM) in the *x*‐direction and a galvanometric mirror (GM) for stepwise scanning in the *y*‐direction, while the laser intensity is modulated synchronously with the focus movement, allowing arbitrary patterns of excitation light to be generated in the focal plane during acquisition of a single camera frame. Here, we focus on sparse fringe patterns with diffraction‐limited fringe width (Figure 1a; Note S1 and Figure S1). The period, position (phase shift), and orientation of the patterns are freely adjustable via the scanning and intensity modulation sequence. Reconstruction of a single SS‐SIM *xy*‐image involves acquisition of a set of raw images (vide infra), all with the same fringe period but different phase shifts and orientations. A 3D image stack is acquired by displacing the sample with a piezo stage in a stepwise fashion along the *z*‐direction, taking raw image sets at each step.

Fringe patterns were simulated for a range of spatial periods, *p*, by convolving the scanned patterns with the computed 3D PSF of our system and integrating the intensity along the axial direction (Note S2; Figure 1b). In addition, we examined our fringe patterns experimentally by mirror reflection in the focal plane of our SS‐SIM system (Note S3 and Figures S2–S6). Both simulation and experiment reveal an increase in fringe visibility (contrast) with period for small periods and subsequent saturation for large periods (Figure 1b; Figure S3). This behavior is to be expected as the fringes are created by scanning a focused spot and, therefore, for greater fringe separations, there is less mutual overlap between main peaks and side lobes of neighboring fringes. In thick biological specimens, scattering from out‐of‐focus regions will also contribute to the intensity detected between the fringes and further deteriorate the contrast. SS‐SIM has a superior optical sectioning capability than conventional SIM, benefiting from an eightfold shorter axial extension of fringe patterns (Figure S4). Notably, the optical sectioning can be further reduced by the sCMOS camera running in confocal slit detection mode [20]. As will be shown below, the high contrast of the sparse fringe patterns used for fluorescence excitation gives rise to the greatly extended imaging depth of SS‐SIM (Figure 1c).

### 2.2 Super‐Resolution Image Acquisition and Reconstruction

**FIGURE 2:**
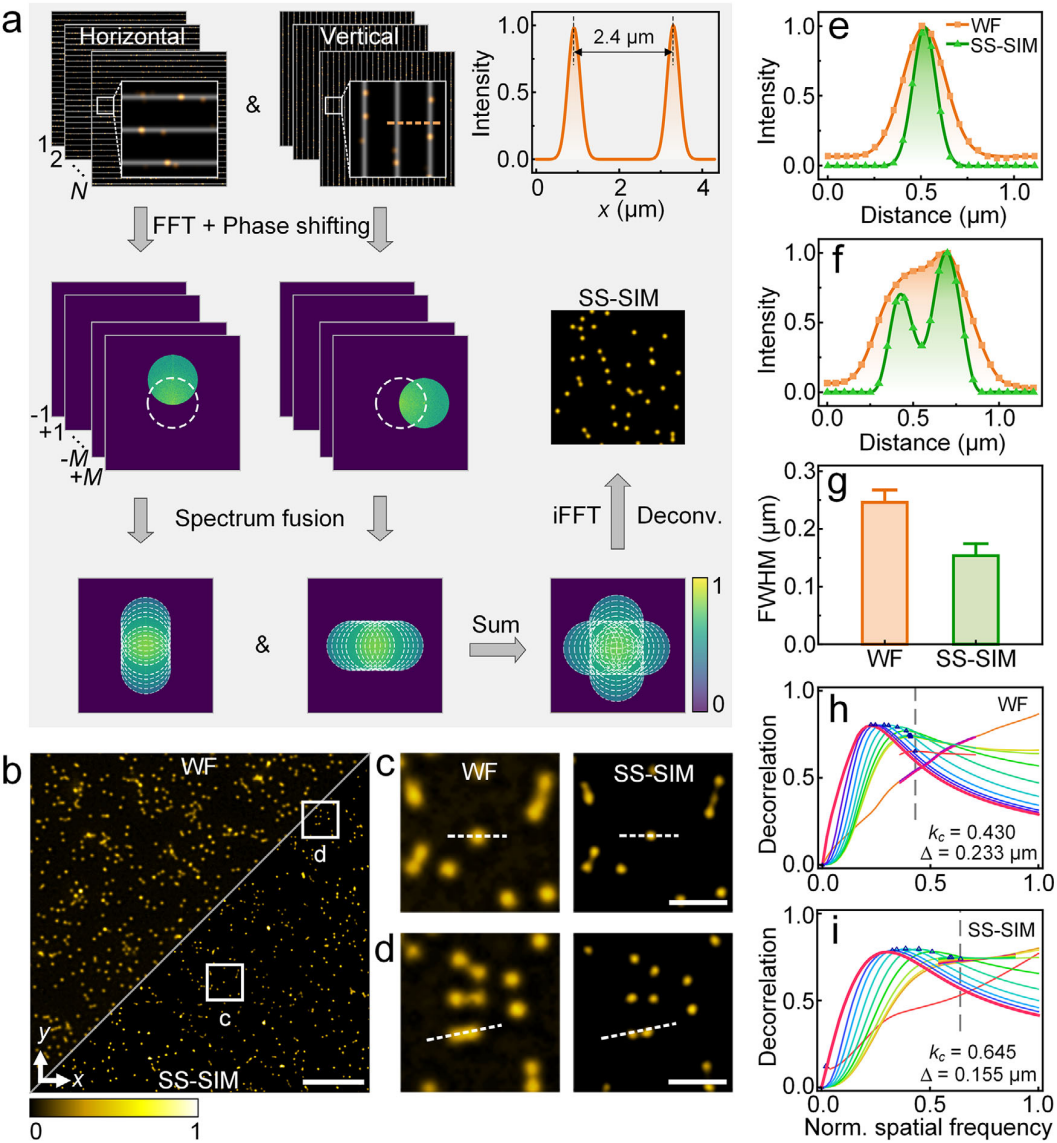
SS‐SIM image reconstruction and resolution estimation based on images of a 2D fluorescent bead phantom. (a) Flow chart of SS‐SIM image reconstruction. The dashed circles indicate spectral regions accessed by harmonics of the sparse fringe pattern. (b) Comparison of WF (upper left) and SS‐SIM (lower right) images of fluorescent microspheres (40 nm diameter). Scale bar, 5 µm. (c,d) WF (left) and SS‐SIM (right) close‐up views of the regions marked by squares in panel b. Scale bar, 1 µm. (e,f) Normalized intensity profiles along the dashed lines marked in panels c and d. Gaussian fits (e) yield FWHMs of 256 ± 4 nm and 162 ± 4 nm for the WF and SS‐SIM modalities. (g) FWHM of the intensity profiles of 400 individual beads chosen by an automatic segmentation algorithm across the entire field of view in panel b. (h,i) Decorrelation‐based resolution analysis of the WF (h) and SS‐SIM (i) images in panel b, resulting in two parameters, the normalized cut‐off frequency, *k_c_* (also indicated by the vertical dashed lines), and the image resolution, Δ, as given in the panels.

The SS‐SIM imaging procedure is shown schematically in Figure 2a. The full width at half maximum (FWHM) of the detection point spread function was determined to be 349 ± 17 nm for 1PE (Figure S5) and 320 ± 17 nm for 2PE (Figure S7), while the fringes generated by focus scanning have an FWHM of 292 ± 14 nm (mean ± s.d.) for 1PE at 640 nm and 424 ± 15 nm (mean ± s.d.) for 2PE at 920 nm. Imaging deep into the sample requires a good fringe contrast, and both simulation (Figure 1b) and experiment (Figure S3) indicate that a fringe visibility as high as ∼0.5 for 1PE (and more for 2PE) is obtained with 2.4‐µm excitation patterns in an ideal sample. Therefore, we set the period of the sparse fringes to 2.4 µm, yielding a good balance between imaging depth and speed (Figure 2a; Figures S3 and S8). For fast imaging, we have implemented an orthogonal illumination patterning scheme [21] exposing the sample to 12 phase‐shifted fringe patterns (in 200‐nm steps) in each of the two lateral dimensions, so that the whole field of view (FOV) is evenly scanned (Figure S6). Thus, for a single SS‐SIM reconstruction, altogether 24 raw images are acquired by the sCMOS camera within 0.65 s (see Methods), which is an order of magnitude faster than with conventional single‐point scanning SIM [22, 23, 24].

Unlike coherent (1PE) SIM featuring sinusoidal fringe patterns, the periodic profiles of our fringe patterns contain five spatial harmonics with significant amplitudes above noise (Note S4 and Figures S9–S11), and image reconstruction akin to the one used for nonlinear SIM [22] allows for an up to ∼1.6‐fold spatial resolution enhancement over standard wide‐field (WF) imaging (see Methods), as anticipated from theoretical estimates for both 1PE and 2PE SS‐SIM (Note S5), and confirmed by our experimental results presented below. For image reconstruction, the 24 raw images are Fourier‐transformed, shifted to their correct positions in frequency space, superimposed, and finally back‐transformed to real space (Figure 2a). Mathematical details are included in Methods.

As an example, Figure 2b shows a combined SS‐SIM (bottom‐right) and WF (top‐left) image of 40‐nm diameter fluorescent microspheres immobilized on a cover slip and excited by 640‐nm light (12 µW in the sample plane). Both parts were rendered from the same dataset; the WF image is simply a superimposition of all 24 raw SS‐SIM images, and deconvolution was performed for both modalities [25, 26]. Two close‐up views (Figure 2c,d) visually attest to the markedly enhanced resolution of SS‐SIM over WF. Intensity profiles across the center of individual bead images show narrower SS‐SIM distributions (Figure 2e), so that two microspheres separated by 255 nm can be clearly resolved (Figure 2f). By using automated segmentation and selecting individual beads to exclude aggregates, we analyzed the FWHM values of 400 beads by fitting their intensity profiles along the *x*‐direction with Gaussians, yielding 246 ± 12 nm (mean ± s.d.) for WF and 154 ± 12 nm (mean ± s.d.) for SS‐SIM (Figure 2g). Since the size of our 40‐nm beads contributes only negligibly to the FWHM, these figures are precise measures of the PSF widths and thus the resolution. Notably, the ratio of 1.6 of the two FWHM values is in excellent agreement with the expected theoretical resolution enhancement factor along the *x*‐coordinate (Note S5). Furthermore, image decorrelation analysis [27] shown in Figure 2h,i, yields normalized cut‐off frequencies, *k_c_* = 0.430 and 0.645, and the corresponding spatial resolutions values [27] Δ  =  pixel size/*k_c_* = 233 and 155 nm, for the WF and SS‐SIM modalities, respectively. Furthermore, the large spectral overlap of the SS‐SIM harmonics in Fourier space is advantageous for background suppression (Note S6 and Figure S12).

### 2.3 Super‐Resolved Volumetric Imaging of a Phantom Sample

**FIGURE 3:**
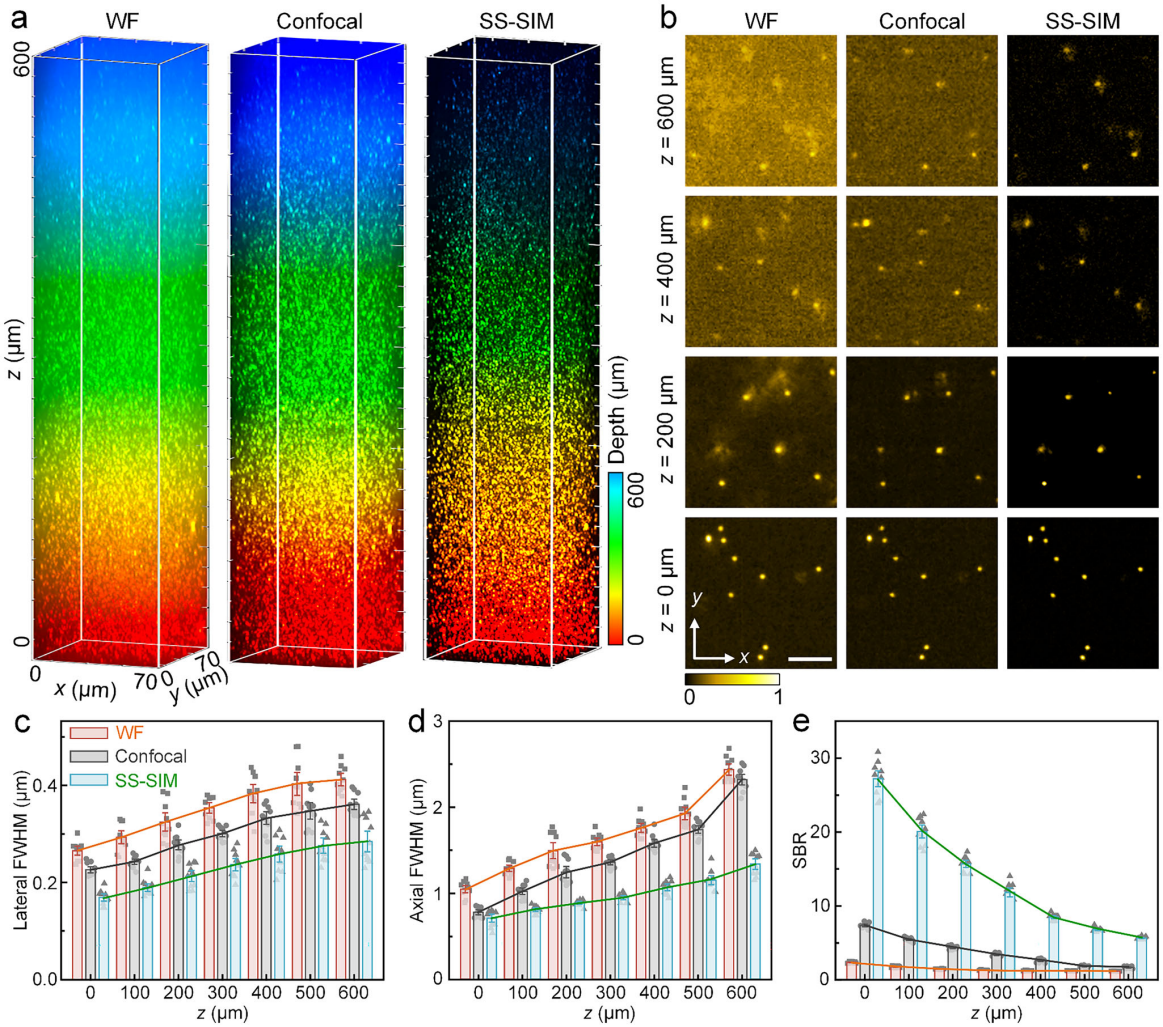
SS‐SIM imaging of a 600 µm thick 3D phantom of fluorescent microspheres immobilized in an agarose gel. (a) 3D image stack of WF (left), confocal (middle), and SS‐SIM (right) images. The color bar on the right encodes the imaging depth. (b) Enlarged views of *xy* cross‐sections at *z* = 0, 200, 400, and 600 µm (bottom to top). The color bar on the bottom encodes intensity; scale bar (applies to all), 5 µm. (c–e) Lateral FWHM (c), axial FWHM (d), and SBR (e) of WF, confocal and SS‐SIM images, determined from ten arbitrarily chosen beads and plotted as a function of imaging depth. The individual bead data are shown as dots, the heights of the bars represent the mean; error bars represent the standard deviation.

To demonstrate deep imaging with enhanced resolution and optical sectioning using SS‐SIM, we prepared a 3D phantom sample by embedding fluorescent microspheres (40 nm diameter) in an agarose gel. We took SS‐SIM 3D image stacks (using our standard protocol) with 640‐nm excitation (1PE, 12 µW in the sample plane) and a long working‐distance objective, taking an image every 400 nm along the axial dimension from *z* = 0 (bottom) to 600 µm (top). WF, line‐confocal (Figure S13), and SS‐SIM images were reconstructed and assembled along the axial direction so as to form a 3D image stack (Figure 3a, Video S1).

Figure 3a shows 3D image stacks with WF, line‐confocal, and SS‐SIM rendering of the same dataset side by side. It is evident that SS‐SIM yields significantly sharper images than WF and line‐confocal over the entire axial range. Moreover, a few bright bead clusters in these samples spread out axially over multiple layers in WF mode due to the lack of axial sectioning. By contrast, they are well captured by SS‐SIM (Figure S14). Selected slices throughout the axial range clearly reveal that SS‐SIM surpasses WF and line‐confocal imaging in regard to resolution and signal‐to‐background ratio (SBR) (Figure 3b).

For quantitative assessment of the imaging performance, we examined the resolution by analyzing FWHMs of fluorescent microspheres as a function of depth. As expected, both lateral and axial resolution deteriorate with imaging depth for all three imaging modalities; however, SS‐SIM maintained a 1.5‐ and 1.3‐fold lateral (Figure 3c), 1.7‐ and 1.4‐fold axial (Figure 3d) resolution enhancement on average over WF and line‐confocal images, respectively. Moreover, there is an impressive SBR enhancement of SS‐SIM, i.e., 8.6‐ and 3.6‐fold, over the WF and line‐confocal modalities (Figure 3e). In summary, we have shown that 1PE SS‐SIM allows for deep 3D super‐resolution imaging within an axial range of up to 600 µm.

### 2.4 Imaging Subcellular Organelles in COS‐7 Cells

**FIGURE 4:**
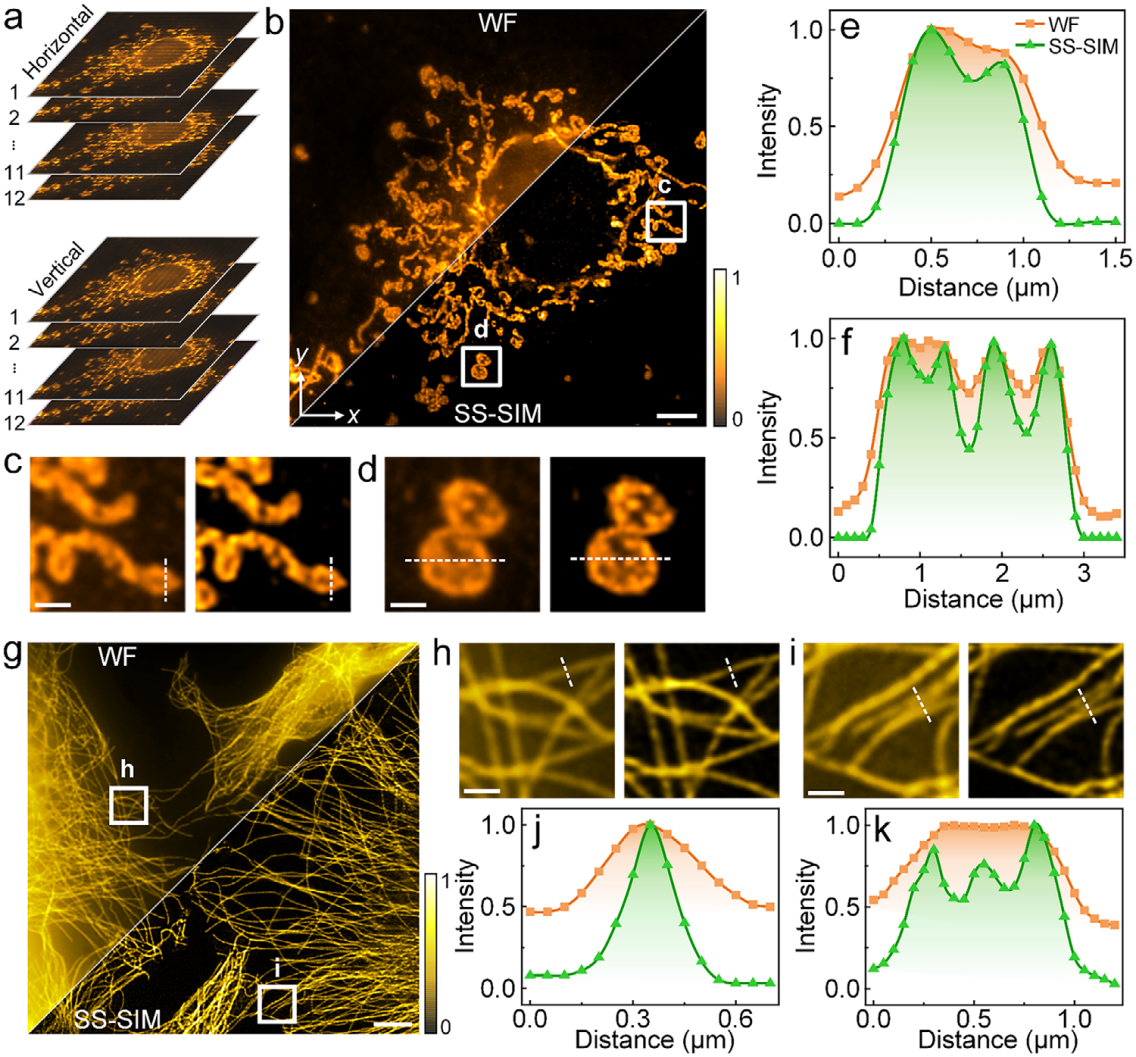
2D SS‐SIM imaging of subcellular structures in fixed COS‐7 cells. (a) Each image is reconstructed from an overall 24 raw images, with twelve phase‐shifted SI patterns each for the horizontal and vertical directions. (b) Comparison of WF (upper left) and SS‐SIM (lower right) images of mitochondria. The color bar on the right encodes intensity; scale bar, 5 µm. (c,d) WF (left) and SS‐SIM (right) close‐up views of the regions marked by squares in panel b; scale bars, 1 µm. (e,f) Normalized intensity profiles along the dashed lines in panels c and d; color assignment to WF and SS‐SIM is shown in panel e (inset). (g) WF (upper left) and SS‐SIM (lower right) images of microtubules. The color bar on the right encodes intensity; scale bar, 5 µm. (h,i) WF (left) and SS‐SIM (right) close‐up views of the regions marked by squares in panel g; scale bars, 1 µm. (j,k) Normalized intensity profiles along the dashed lines marked in panels h, i; color assignment to WF and SS‐SIM is shown in panel e (inset).

Next, we examined the performance of SS‐SIM for imaging subcellular structures of cultured COS‐7 cells (simian fibroblast‐like cell line), using 640‐nm excitation (1PE, 60 µW in the sample plane). To this end, COS‐7 cells were fixed and immunolabeled with antibodies against Tom20, a receptor subunit located in the outer mitochondrial membrane, so that mitochondria can be visualized selectively. Figure 4a shows schematically the 12 raw images in each of the two fringe orientations that were acquired for SS‐SIM (and WF) image reconstruction. The enhanced resolution and image contrast of SS‐SIM over WF imaging is clearly visible in the combined WF/SS‐SIM image (Figure 4b), and also in two close‐up images displaying hollow structures inside elongated mitochondria (Figure 4c) as well as granular nodules in spherical mitochondria (Figure 4d), which are blurred in the WF images. Normalized intensity profiles further support these findings in a more quantitative fashion (Figure 4e,f).

Next, we immunolabeled fixed COS‐7 cells with antibodies against α‐tubulin for visualizing microtubules, long and hollow cytoskeletal fibers of 25 nm diameter. Images were collected as described above. The combined reconstructed image (Figure 4g) again demonstrates the higher image resolution and SBR of SS‐SIM over WF. Figure 4h,i displays close‐up WF (left) and SS‐SIM (right) images of the two regions marked in Figure 4g. Gaussian fitting of intensity profiles (Figure 4j) across a microtubule yields FWHMs of 275 ± 14 nm in WF and 171 ± 6 nm in SS‐SIM mode, confirming 1.6‐fold resolution enhancement of SS‐SIM over WF, and the SBR was fivefold enhanced in SS‐SIM. Figure 4k gives an example where SS‐SIM clearly resolves three closely spaced microtubules that are indistinguishable in WF images (Figure 4i).

### 2.5 Volumetric Imaging of Zebrafish Larvae

**FIGURE 5:**
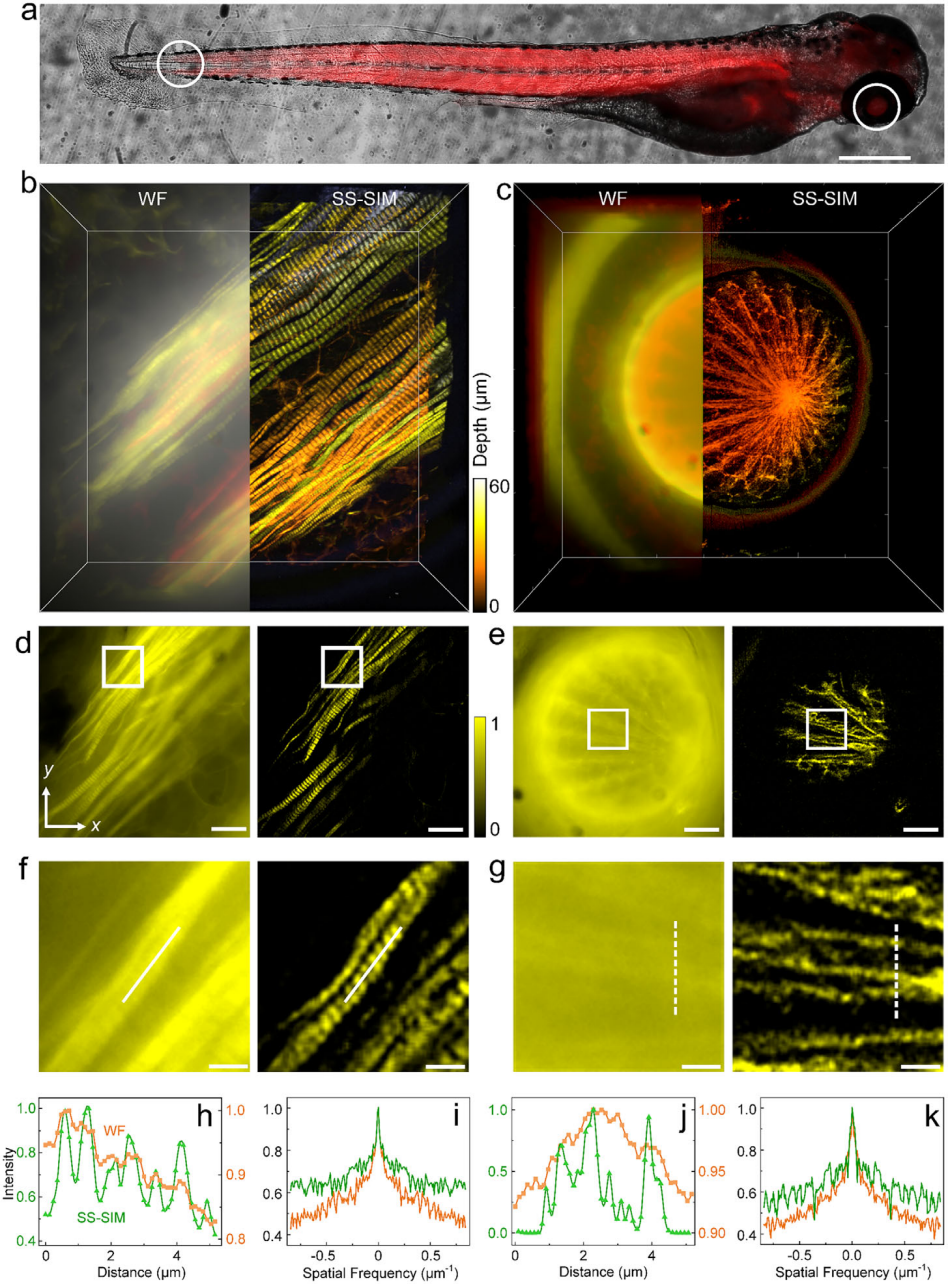
3D SS‐SIM imaging of fixed zebrafish larvae in a depth range up to 60 µm. (a) Overlap of bright‐field and fluorescence images of a zebrafish with STAR RED phalloidin‐labeled actin; scale bar, 300 µm. (b,c) 3D WF and SS‐SIM close‐up views of the regions indicated by circles in panel a (tail: panel b; eye: panel c). Color bar (applies to b, c), depth encoding; volume, 140 × 140 × 60 µm^3^. (d,e) Comparison of WF (left) and SS‐SIM (right) images of 2D slices through the 3D images in panels b and c at *z* = 19 and 13 µm, respectively. Scale bars, 10 µm. (f,g) Enlarged views of the regions marked by squares in panels d and e. Color bar (applies to d‐g), intensity encoding; scale bars, 2 µm. (h) Normalized intensity profiles along the dashed lines of panel f; colors (applying to panels h‐k) assigned to WF and SS‐SIM modes are shown in the graph. (i) Normalized spectrum of the image in panel f along the diagonal. (j) Normalized intensity profiles along the dashed lines in panel g. (k) Normalized spectrum of the image in panel g along the diagonal.

Zebrafish has been established as an excellent system for whole‐organism imaging. Here, we chose zebrafish larvae as thick specimens for deep tissue SS‐SIM imaging with 1PE. 72 h post fertilization (hpf), larvae were fixed and labeled with a conjugate of the bright fluorophore STAR RED (peak excitation/emission at 638/655 nm) and phalloidin, a cyclic heptapeptide from *Amanita phalloides* (death cap) mushrooms, which strongly binds to and stabilizes filamentous actin (F‐actin). Therefore, its conjugates with fluorophores are widely used to label actin filaments for imaging. Figure 5a shows an overlay of a bright‐field and a WF fluorescence image of a zebrafish larva. We selected two subvolumes (140 × 140 × 60 µm^3^), located at the tail (Figure 5b) and the eye (Figure 5c) of the larva, for collecting 3D SS‐SIM image stacks. With an axial step size of 200 nm, 300 SS‐SIM (and WF) images were reconstructed from 7200 raw images.

In the WF image of the tail region, the muscle fibers are poorly resolved and almost indistinguishable from the high background noise (Figure 5b; Video S2). By contrast, the SS‐SIM image is essentially free of background, and the enhanced resolution allows for clear visualization of the cross‐striations of the tail muscle fibers due to selective F‐actin labeling of thin filaments (Figure 5b), featuring a FWHM of 293 ± 32 nm (mean ± s.d.). WF and SS‐SIM images of a single layer at *z* = 19 µm and close‐up views (Figure 5d,f) further confirm the effective background suppression of SS‐SIM due to its excellent optical sectioning capability. The normalized intensity distributions along the dashed lines in Figure 5f demonstrate the enhanced resolution of SS‐SIM over the WF modality (Figure 5h), as is also indicated by the different proportions of high‐frequency components in the corresponding spectra (Figure 5i).

In the zebrafish eye, SS‐SIM allows us to clearly visualize the radially distributed pupillary dilator muscle fibers of the iris (Figure 5c,e,g,j,k; Video S3), which is responsible for pupil widening. By contrast, the WF image is drowned in the background due to the lack of optical sectioning. Comparison with standard confocal laser scanning microscopy (CLSM) further revealed that SS‐SIM achieves a similar penetration depth and SBR as CLSM, however, with a 1.6‐fold enhanced lateral resolution and 67‐fold faster image acquisition (Note S7 and Figure S15).

### 2.6 Volumetric Imaging With Two‐Photon Fluorescence Excitation

2PE fluorescence microscopy is based on the simultaneous absorption of two low‐energy near‐infrared (NIR) photons for exciting fluorophores in the visible range of wavelengths. This process occurs with appreciable yields in regions of very high spatio‐temporal photon density. 2PE transitions can be induced effectively by tight focusing of a pulsed laser beam and occur only near the focal center, providing optical sectioning and avoiding photobleaching outside the focal spot. Furthermore, the use of long‐wavelength NIR light is beneficial due to its reduced scattering. These properties have rendered 2PE fluorescence microscopy a superb imaging modality for deep imaging into tissues. As fringe patterns in SS‐SIM are generated by beam scanning, 2PE can be easily implemented in an SS‐SIM system. In our system, we have simply included a 920‐nm femtosecond pulsed laser as a 2PE excitation source.

**FIGURE 6:**
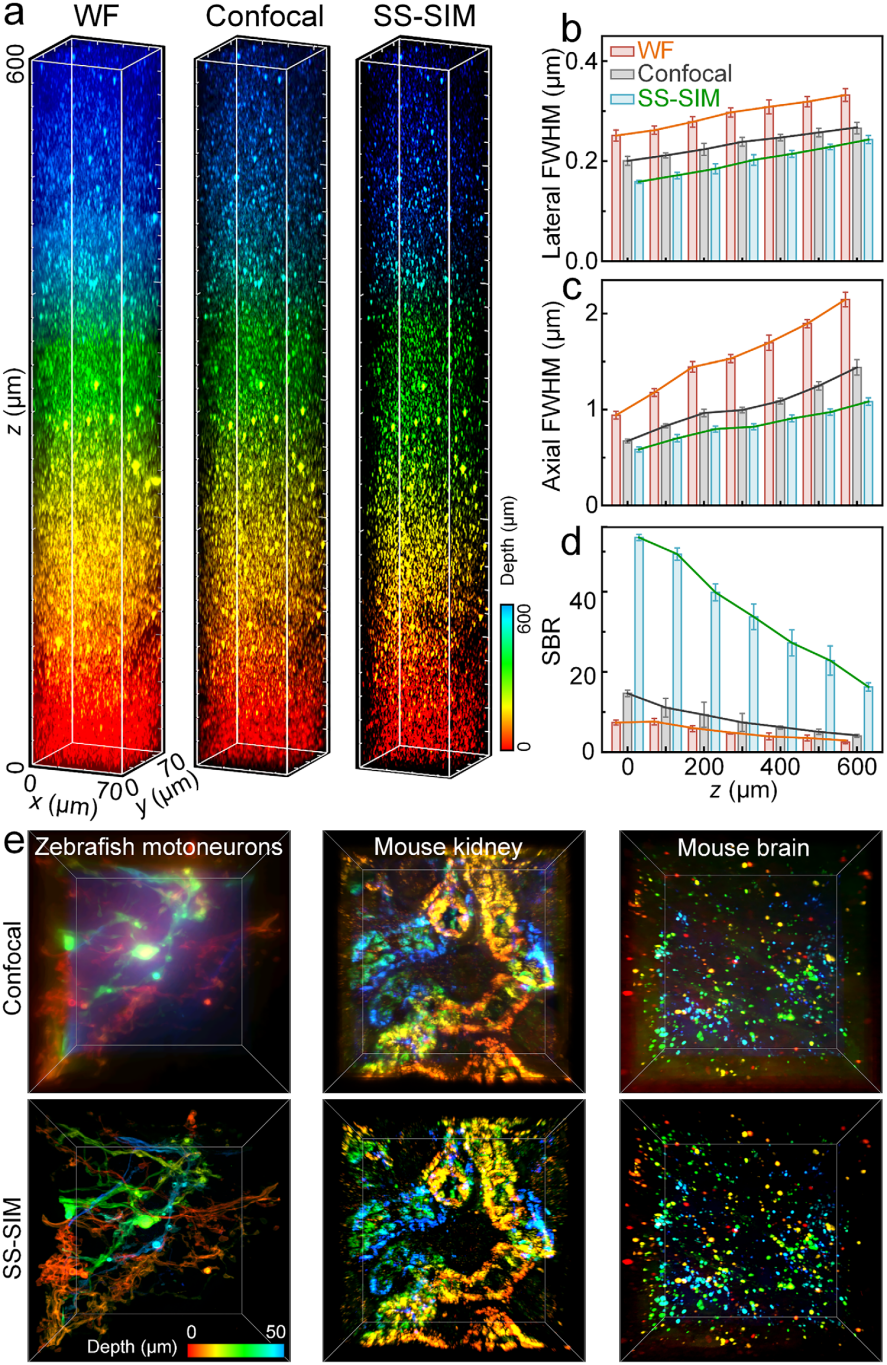
3D SS‐SIM with 2PE. (a) Volumetric images (70 × 70 × 600 µm^3^) of a phantom consisting of fluorescent microspheres (40 nm diameter) immobilized in an agarose gel, taken with WF (left), confocal (middle), and SS‐SIM (right) modalities. (b–d) Lateral FWHM (b), axial FWHM (c), and SBR (d) of WF, confocal, and SS‐SIM images, determined from ten arbitrarily chosen beads and plotted as a function of imaging depth. The heights of the bars represent the mean; error bars represent the standard deviation. (e) Confocal (upper) and SS‐SIM (lower) volumetric images (70 × 70 × 50 µm^3^) of different biological tissues. From left to right: GFP‐labeled zebrafish motoneurons, ZsGreen‐labeled mouse kidney and brain sections. Color bar, depth encoding.

We examined the SS‐SIM performance with a phantom sample consisting of 40‐nm fluorescent microspheres in an agarose gel. By using a long working‐distance objective with *NA* = 1.1, a volume of 70 × 70 × 600 µm^3^ was imaged with 35‐mW laser power in axial steps of 400 nm, thus altogether 1500 images were reconstructed from 36 000 raw images. Figure 6a shows 3D reconstructions from WF, line‐confocal (Note S8 and Figure S13), and SS‐SIM modalities, all based on the same image dataset and processed by a balanced deconvolution. In all three modalities, we observe punctate features throughout the entire depth range. However, in comparison to the WF and confocal modalities, SS‐SIM offers a markedly higher image quality, with 1.5‐ and 1.2‐fold enhanced lateral resolution, 1.8‐ and 1.2‐fold enhanced axial resolution, and 4.3‐ and 7.5‐fold greater SBR, respectively (Figure 6b–d). Notably, with our sparse illumination patterns, we achieve eightfold greater depth than a recent study using 2PE scanning SIM [12].

We further employed 2PE SS‐SIM for imaging various densely structured biological specimens prepared from transgenic animals expressing fluorescent proteins of the GFP family, which are powerful labeling tools for live organismal imaging [28, 29]. These include GFP‐labeled zebrafish (line *Tg(mnx1:mGFP)* [30]) motoneurons (Video S4) and ZsGreen‐labeled mouse (line C57BL/6JGpt‐H11^em1Cin(^ *^CAG‐LoxP‐ZsGreen‐Stop‐LoxP‐tdTomato^* ^)^/Gpt) kidney and brain slices (Figure 6e). With ∼50 mW of 920‐nm laser power, we imaged volumes of 70 × 70 × 50 µm^3^ with an axial step size of 200 nm. For all three samples, visual inspection shows that the SS‐SIM images have superior SBR and spatial resolution over the line‐confocal images (Figure 6e). Decorrelation and spectral analysis on three sets of images revealed a 1.5‐fold enhanced resolution and a 3.7‐fold greater SBR of 2PE SS‐SIM over the line‐confocal modality.

**FIGURE 7:**
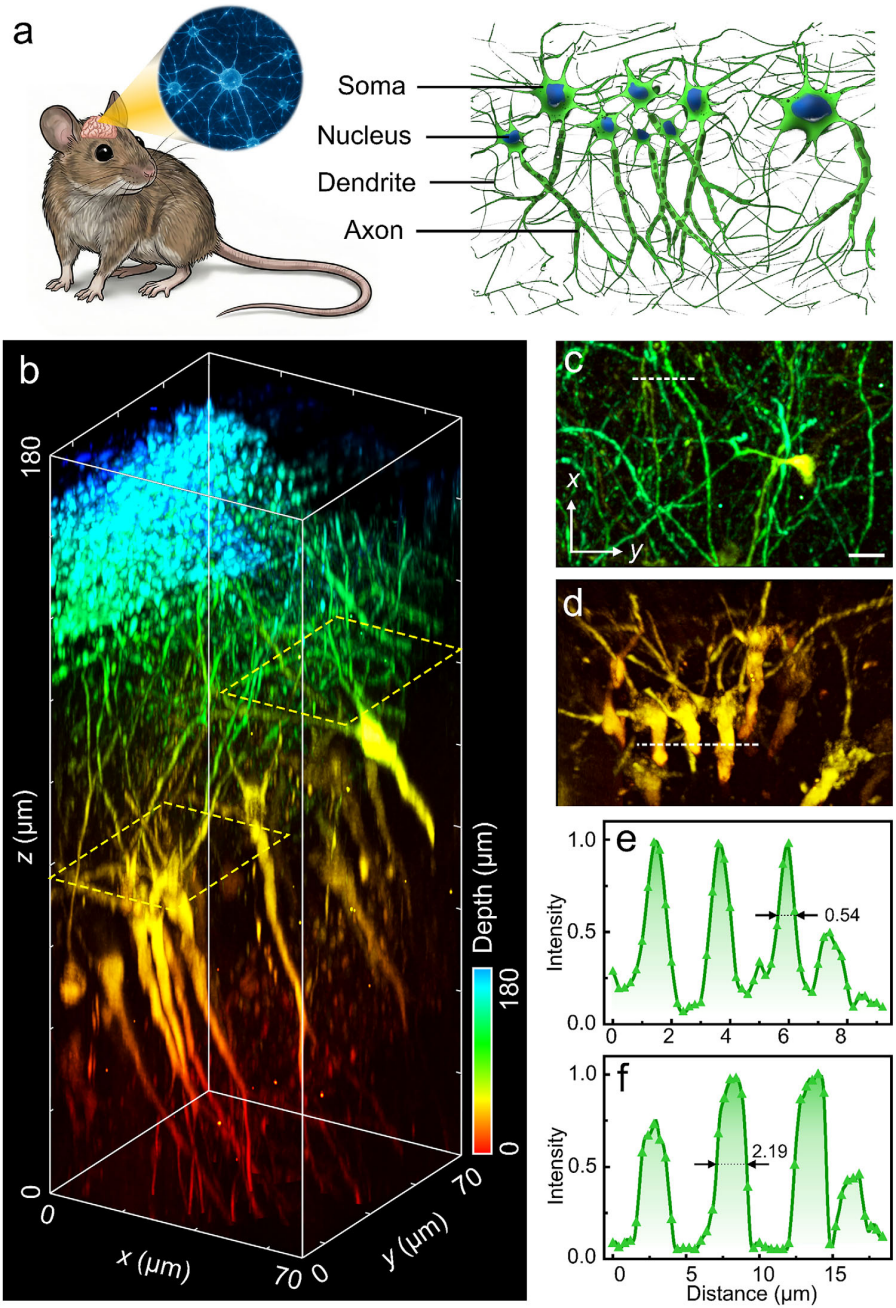
3D imaging of Thy1‐EGFP mouse brain slices by 2PE SS‐SIM. (a) Schematic diagram of neurons in the mouse brain. (b) Reconstructed 3D SS‐SIM image stack of a region of a mouse brain slice (70 × 70 × 180 µm^3^). The color bar indicates depth encoding. (c,d) Enlarged views of *xy*‐sections at *z* = 124 (c) and 76 µm (d), indicated by the yellow lines in panel b. The 35 × 53 µm^2^ sections were computed by overlaying the intensities of *z*‐layers in an overall range of 15 µm around the axial depth. Scale bar (applies to all), 5 µm; the color bar is the same as in panel b. (e,f) Normalized intensity profiles along the dashed lines in panels c and d. Gaussian fits yield FWHMs of 0.54 ± 0.02 µm for a dendrite (e) and 2.19 ± 0.11 µm for a cell body (f).

Finally, we examined cleared brain tissues from mice expressing Thy1‐EGFP fusion proteins in their neurons (Figure 7a). Again with 50 mW of 920‐nm laser power, we imaged volumes of 70 × 70 × 180 µm^3^ with an axial step size of 200 nm. The 3D SS‐SIM image clearly displays cell bodies, axons, and dendrites of the neuronal network (Figure 7b; Video S5). The *xy*‐ sections at *z* = 76 and 124 µm clearly reveal detailed structures of neuronal dendrites (Figure 7c) and cell bodies (Figure 7d), and the intensity profiles in Figure 7e,f show typical FWHM values of 0.54 ± 0.02 µm and 2.19 ± 0.11 µm, respectively. Furthermore, a quantitative comparison with 1PE CSLM yields a roughly 6‐fold higher SBR for SS‐SIM at an imaging depth of 100 µm (Figure S16). Taken together, our tissue imaging experiments demonstrate that 2PE SS‐SIM allows for imaging thick samples with an enhanced SBR and spatial resolution over WF and confocal imaging modalities.

## 3 Discussion

In this work, we demonstrate that SS‐SIM enables fluorescence imaging of structures deep within thick specimens with high contrast and spatial resolution in both lateral and axial dimensions. The optical design is conceptually straightforward, combining rapid focused laser beam scanning with pixel‐addressed intensity modulation and synchronized sCMOS detection. Owing to this simplicity, SS‐SIM can be integrated into existing confocal microscope platforms with only moderate modification. Notably, we employed water‐immersion objectives with intermediate numerical apertures (*NA* 1.1/1.2), rather than high‐*NA* oil objectives, to ensure compatibility with live‐specimen imaging.

In our current SS‐SIM implementation, five harmonics contribute to the periodic fringe pattern, yielding ∼1.6‐fold lateral and ∼1.7‐fold axial resolution enhancement beyond the diffraction limit. The use of highly focused light for structured illumination gives rise to the method's excellent optical sectioning capability [31]. Ultimately, image resolution is constrained by the width of the scanned focus, which can be further sharpened through radially polarized light [32, 33] or beam shaping [34]. Potential focal side lobes can be effectively suppressed owing to the large fringe separation intrinsic to SS‐SIM, either by the camera (via confocal slit detection) or through image post‐processing. In future work, we will investigate structured fluorescence excitation using 2D lattice patterns instead of 1D fringes. Orthogonal [35] and triangular [36] lattices reduce the required number of raw images from 24 to five and seven, respectively, thereby accelerating data acquisition. A further advantage of 2D lattices is that they eliminate the need for pattern re‐orientation and polarization matching to ensure optimal fringe visibility.

The orthogonal line‐scanning strategy employed in SS‐SIM shares conceptual similarities with other high‐speed, deep tissue‐imaging modalities [17, 37, 38, 39]. Among these, line‐scan SIM [17] and multiline orthogonal scanning temporal focusing (mosTF) microscopy [38] are restricted in imaging speed due to slow linear scanning. Two‐photon instant SIM (2P ISIM) [37] enables real‐time super‐resolution imaging yet lacks background suppression via phase shifting of fringe patterns. Multi‐confocal ISM (MC‐ISM) [39] employs multi‐focus scanning and enables super‐resolved imaging of thick specimens with high speed and low photodamage. Still, multiplexing several foci subdivides the full aperture, which inevitably reduces the achievable axial depth. Compared with all these imaging modalities, our SS‐SIM method emerges as a powerful tool for rapid super‐resolution imaging of rather thick specimens (Table S1). It excels in imaging depth, owing to the sparse fringe patterns generated with tightly focused illumination, and its high imaging speed primarily originates from fast resonant beam scanning synchronized with laser intensity modulation.

The use of focused excitation renders SS‐SIM particularly well‐suited for multiphoton imaging, and multiple time‐modulated lasers enable multi‐color fluorescence imaging, including combinations of 1PE and 2PE. Simultaneous imaging of selected regions of interest within the field of view—for example, cellular organelles—in distinct colors and excitation modes also appears feasible. Moreover, integration of SS‐SIM with second‐harmonic generation (SHG) imaging is both attractive and straightforward, although SHG, as a coherent imaging modality, does not benefit from resolution enhancement [40].

Our current SS‐SIM implementation features sparse fringe patterns with a fixed spatial period (2.4 µm). In the future, we will implement a scanning scheme featuring a fringe period that increases with depth. This approach ensures, on the one hand, fast imaging by using a small fringe period at smaller depths and, on the other hand, an enhanced signal‐to‐noise ratio by using a larger fringe period at larger depths. Furthermore, we will augment our SS‐SIM system with adaptive optics to compensate for system‐ and sample‐induced aberrations [41, 42, 43]. Such aberrations can substantially distort the focal spot at increased imaging depths and thereby reduce image resolution and SBR, especially in thick and strongly scattering samples. Aberrations can be probed by using embedded luminescent guide stars or by analyzing the scanned fringe patterns in raw images, and dynamic optical elements such as deformable mirrors (DMs) or spatial light modulators (SLMs) in the illumination path allow for precise pre‐distortion of the wavefronts in real time to compensate for the aberrations. Obviously, data acquisition will be a bit slower with aberration correction.

In conclusion, we have demonstrated that SS‐SIM provides significant advantages for deep‐tissue fluorescence imaging using either 1PE or 2PE. Owing to its conceptual simplicity, we anticipate that SS‐SIM will emerge as a versatile tool for high‐resolution imaging of tissues, organoids, and model organisms, including live specimens.

## 4 Methods

### 4.1 Microscope Setup

A schematic diagram of the SS‐SIM apparatus is shown in Figure 1a. A 640‐nm fiber‐coupled diode laser (iBEAM smart 640‐S, 150 mW, TOPTICA, Germany) delivering pulses at a rate of 250 MHz is employed for (linear) fluorescence excitation. Alternatively, we use a 920‐nm femtosecond pulsed NIR laser (FemtoNL‐920 nm, 1 W, 200 fs, 80 MHz, Wuhan Yangtze Soton Laser Co., Ltd, China) for 2PE. Light exiting the fiber is collimated by lens L1 (f = 30 mm), and the parallel beam is scanned by a 2D galvo‐resonant scanner (LSK‐GR12/M, Thorlabs, USA) combining a fast resonant scanning mirror (RM) oscillating at 12 kHz with a slower regular galvo mirror (GM). A telescope system consisting of scan lens SL (f = 100 mm) and tube lens TL1 (f = 200 mm) relays the scanning plane (halfway between RM and GM) to the back pupil plane of the objective, ensuring that the beam can enter the objective pupil under all deflection angles. A microscope objective (MO, HC PL APO 63× NA 1.2, water immersion; HC PL IRAPO 40× NA 1.1, water immersion, Leica, Germany) focuses the beam into a tight spot that is steered by the 2D scanner across the *xy*‐plane. Notably, the 40× objective has a long working distance and was used for imaging at a depth up to 600 µm (Figures 3a and 6a). A piezo stage (SLR‐800, Piezoconcept, France) allows for sample scanning along the axial direction for 3D imaging.

The fluorescence emitted by the sample is collected by the objective lens, passed through a quad‐band dichroic mirror (DM, 1PE: Di03‐R405/488/561/635‐t3‐25×36, 2PE: FF880‐SDi01‐t1‐25×36, Semrock, USA) and an emission filter (EF, 1PE: FF01‐446/523/600/677‐25, 2PE: FF01‐433/530‐25, Semrock, USA), and focused by a second tube lens (TL2, f = 200 mm) onto an sCMOS camera (Orca‐Flash4.0 LT, Hamamatsu, Japan). The 2D scanner (RM/GM), axial sample stage, laser power modulation, and image acquisition by the sCMOS camera are synchronized by a data acquisition board (DAQ, USB6363, National Instruments, USA) and a personal computer (PC) running a custom LabVIEW program, as described in detail in Note S1.

### 4.2 Beam Scanning for SS‐SIM Imaging

Sparse fringe patterns are generated by scanning the laser spot in synchrony with laser power modulation (Note S1 and Figure S1a–c). Performing the fast resonant scan along the *x‐*direction and the linear scan in the *y*‐direction, we can generate arbitrary cumulative intensity patterns, *I*(*x*,*y*), in the focal plane by switching the laser power on at the desired focus positions, *x*(*t*) and *y*(*t*). Maintaining a balance between the imaging speed and the resolution enhancement in SS‐SIM, we have chosen to generate sparse fringe patterns along the *x‐* and *y*‐directions, with periods of 2.4 µm, to achieve high contrast (Figure 1b) and twelve 200‐nm steps for phase shifting. Therefore, altogether 24 raw images are acquired by the sCMOS camera for a single SS‐SIM image reconstruction.

To create patterns with fringes oriented along the *y‐*direction, 200 lines are written by the fast‐scanning RM, each displaced by 0.35 µm, which is roughly the 1/e width of the focal spot, along the *y‐*direction by the GM. During a single fast scan, the laser is switched on briefly in suitable time intervals to write spots equally spaced by 2.4 µm along the *x‐*direction (Figure S1d). Notably, forward and backward RM scanning phases are overlapped in the raw image. In this mode, scanning a FOV of 70 × 70 µm^2^, which is sampled by 700 × 700 pixels on the camera, takes 200 × 83.33 µs ≈ 17 ms. To create patterns with fringes oriented along the *x‐*direction, 29 RM lines are written, each displaced by 2.4 µm along the *y*‐direction by the GM. During a single fast scan, the laser is on all the time (Figure S1e). Each RM scan is repeated seven times, so that the generation of the *x*‐ and *y*‐oriented fringe patterns takes the same time (17 ms). Including the camera readout time of 10 ms for each raw image, the 24 raw images needed for a full SS‐SIM reconstruction are captured in 0.65 s.

### 4.3 Microscope Adjustment and Fringe Pattern Characterization

A precise overlay of the focused fringe pattern with the focal plane of detection is of utmost importance for SS‐SIM. Therefore, we adjust our microscope using a 2D scale reticle (Note S3 and Figure S2). For characterization of the fringe patterns, a planar, silver‐coated mirror is positioned in the focal plane of the objective lens by varying the distance between the mirror and the MO to yield the thinnest fringes, and the emission filter EF was removed. The widths of the fringes in the *x‐* and *y*‐orientations are examined by analyzing the fringe images using Gaussian fitting of the intensity profiles to obtain the FWHM values (Figure S5). Furthermore, phase‐shifting of the fringe patterns in steps of 200 nm is confirmed by direct imaging of the patterns (Figure S6).

### 4.4 SS‐SIM Image Reconstruction and Processing

The intensity pattern imprinted on the sample is a picket‐fence arrangement of sharp spikes with diffraction‐limited widths. Thus, it is periodic yet anharmonic, and can be represented by a Fourier series,

(1) $$I_{n}\;\left(r\right)=\sum_{m=1}^{M}2I_{m}\left[1+\cos\left(2\pi mk_{p}r+m\varphi_{n}+\varphi_{0m}\right)\right]$$

here, *I_n_* (*r*) is the intensity of the pattern as a function of the spatial coordinate, *r*, oriented perpendicular to the direction of the fringes, the subscript *n* enumerates the number of phase shift steps up to a maximum of *N*. The series is expressed as a sum of cosine functions with amplitudes, 2*I_m_*, where *m* indicates the order of the harmonics up to *M*. The argument of the cosine functions contains the fundamental spatial frequency of the fringe pattern, *k_p_* =  1/*p*, *p* being the period of the fringe pattern, the phase shift for the *n*‐th shifting step, φ_*n*_ =  2π*n*/*N*, and a constant phase offset for each of the harmonics, φ_0*m*_.

Upon excitation with this pattern, the fluorescence image detected by the sCMOS camera is given by

(2) $$D_{n}\;\left(r\right)=\left[I_{n}\left(r\right)\cdot S\left(r\right)+b\left(r\right)\right]\;\;⊗\;PSF\left(r\right)$$

here, *S*(*r*) represents the spatial distribution of fluorophores in the sample, which is multiplied and thus modulated by the intensity pattern, *I_n_* (*r*), and *b*(*r*) denotes a spatially dependent background that includes sample scattering and detector noise. In the microscope, this ideal image is convolved (‘⊗’) with *PSF*(*r*), the point spread function of the system. For image reconstruction, we use the Fourier space representation of Equation (2),

(3) $${\tilde{D}}_{n}\;\left(k\right)=\left[{\tilde{I}}_{n}\left(k\right)\;⊗\;\tilde{S}\left(k\right)+\tilde{b}\left(k\right)\right]\;\cdot OTF\left(k\right)$$

where *k* is the spatial frequency, ${\tilde{D}}_{n}\left(k\right)$, ${\tilde{I}}_{n}\left(k\right)$, $\tilde{S}\left(k\right)$ and $\tilde{b}\left(k\right)$ are the Fourier transforms of *D_n_* (*r*), *I_n_* (*r*), *S*(*r*) and *b*(*r*), respectively, and the optical transfer function (OTF), *OTF*(*k*), of the system is the Fourier transform of *PSF*(*r*).

To extract the contributions of the different harmonics to the image requires at least *N* = 2*M* + 1 independent equations of the type of Equation (3). In matrix notation, the system of equations can be written as

(4) $$\begin{array}{ccc} \left(\begin{array}{c} {\tilde{D}}_{1}\\ {\tilde{D}}_{2}\\ {\tilde{D}}_{3}\\ \vdots \\ {\tilde{D}}_{N} \end{array}\right) & = & \left(\begin{array}{cccccc} 1 & I_{1}e^{i\varphi_{1}} & I_{1}e^{-i\varphi_{1}} & \text{…} & I_{M}e^{iM\varphi_{1}\;} & I_{M}e^{-iM\varphi_{1}\;}\\ 1 & I_{1}e^{i\varphi_{2}} & I_{1}e^{-i\varphi_{2}} & \text{…} & I_{M}e^{iM\varphi_{2}\;} & I_{M}e^{-iM\varphi_{2}\;}\\ 1 & I_{1}e^{i\varphi_{3}} & I_{1}e^{-i\varphi_{3}} & \text{…} & I_{M}e^{iM\varphi_{3}\;} & I_{M}e^{-iM\varphi_{3}\;}\\ \vdots & \vdots & \vdots & \vdots & \vdots & \vdots \\ 1 & I_{1}e^{i\varphi_{N}} & I_{1}e^{-i\varphi_{N}} & \text{…} & I_{M}e^{iM\varphi_{N}\;} & I_{M}e^{-iM\varphi_{N}\;} \end{array}\right)\\ & & \times \left(\begin{array}{c} \tilde{W_{f}}\left(k\right)\\ e^{i\varphi_{01}}\tilde{S_{f}}\left(k-k_{p}\right)\\ e^{-i\varphi_{01}}\tilde{S_{f}}\left(k+k_{p}\right)\\ \vdots \\ e^{i\varphi_{0M}}{\tilde{S}}_{f}\left(k-Mk_{p}\right)\\ e^{-i\varphi_{0M}}\tilde{S_{f}}\left(k+Mk_{p}\right) \end{array}\right) \end{array}$$

here, $\tilde{W_{f}}\left(k\right)$, $\tilde{S_{f}}\left(k\pm k_{p}\right)$, $\tilde{S_{f}}\left(k\pm 2k_{p}\right)$, ⋅⋅⋅ $\tilde{S_{f}}\left(k\pm Mk_{p}\right)$ are the different image components low‐pass filtered by the OTF of the system. By solving this system of equations, image components with different frequency shifts, $\tilde{W_{f}}\left(k\right)$, $\tilde{S_{f}}\left(k\pm k_{p}\right)$, $\tilde{S_{f}}\left(k\pm 2k_{p}\right)$, ⋅⋅⋅, and $\tilde{S_{f}}\left(k\pm Mk_{p}\right)$ are isolated. In the image reconstruction, their displacement by multiples of *k_p_* in frequency space is removed and, finally, their superimposition is Fourier‐transformed to reconstruct a real‐space image with enhanced resolution. Notably, $\tilde{W_{f}}\;\left(k\right)=\sum_{m=1}^{M}2I_{m}\;\tilde{S_{f}}\left(k\right)+\tilde{b_{f}}\left(k\right)$, carrying an image content equivalent to that of conventional widefield microscopy under epi‐illumination, is invariant under phase shifting. Therefore, by selectively omitting $\tilde{W_{f}}\left(k\right)$ from the image synthesis, only in‐focus fluorescence contributions are included, endowing SS‐SIM with remarkable optical sectioning capability.

The number of harmonics is governed by the logarithm of the ratio between the fringe period and width. In our setup, we are limited to *M*  =  5 for both 1PE and 2PE (Figures S10 and S11). Thus, at least *N* = 2*M* + 1 = 11 phase‐shifted images are needed for reconstruction in each fringe orientation. Here, we chose *N* = 12 phase shifts to ensure the step size (200 nm) is perfectly sampled with two pixels on the sCMOS camera sensor when using a water immersion objective with 63× magnification. Furthermore, the final images for all imaging modalities used in this work (WF, confocal, and SS‐SIM) were treated with a balanced deconvolution [25, 26]. 3D image stacks were assembled from 2D images by proper positioning along the axial direction. We also examined the joint Richardson‐Lucy deconvolution algorithm [44] for reconstruction of SS‐SIM images, including a test with reduced numbers of raw images (Figure S17). However, for achieving an equivalent spatial resolution enhancement, the algorithm turned out to be computationally much more expensive.

### 4.5 Preparation of Single‐Layer Fluorescent Microsphere Samples

A cleaned circular coverslip (18 mm diameter) was incubated with 150 µL of poly‐L‐lysine (P4707, Sigma‐Aldrich, USA) for 30 min and then rinsed thrice with deionized water. A stock solution of fluorescent microspheres (diameter 40 nm, 1PE: F8789, 660/680 nm; 2PE: F8766, 505/515 nm, Invitrogen, USA) was diluted 1:10^4^ in phosphate‐buffered saline (PBS) (G4202, Servicebio, China) to obtain a sparse decoration of the glass surface with microbeads. 150 µL of the solution was applied to the coverslip surface and left to adhere for 30 min. The coverslip was again rinsed thrice with deionized water to remove loosely adhered microspheres, covered with a cleaned glass slide using 25 µL of ProLong Glass Antifade Mountant (P36984, Invitrogen, USA). To allow for curing, the assembly was kept at room temperature (RT) for 1 h prior to imaging.

### 4.6 Preparation of Agarose Gel Samples With Immobilized Fluorescent Beads

A stock solution of fluorescent microspheres (1PE: F8789, 660/680 nm; 2PE: F8766, 505/515 nm, Invitrogen, USA) was diluted to 1:10^4^ in PBS. After adding 8 mg of agarose powder (A9045, Sigma‐Aldrich, USA) to 400 µL of the solution, the mixture was heated to 69 °C in a thermostatic bath (MDB100, JOANLAB, China) to ensure proper dissolution of the agarose. During heating, the mixture was vortexed to blend evenly. Subsequently, the mixture was transferred into a well of an 8‐chambered cover glass (C8‐1.5H‐N, Cellvis, USA). By cooling in a refrigerator, the sample was gelled within 10 min and ready for use.

### 4.7 Culturing and Immunofluorescence Labeling of COS‐7 Cells

COS‐7 cells were grown in DMEM (SH30022.01, HyClone, USA) supplemented with 10% (v/v) fetal bovine serum (FBS, SV30208.02, HyClone, USA) and 1% (v/v) penicillin‐streptomycin (PS, G4003, Servicebio, China) in a humidified atmosphere containing 5% CO_2_ at 37 °C. They were seeded on clean coverslips 24 h prior to fixation to reach a confluency of ∼70%.

For immunofluorescence labeling, mitochondria and microtubules were tagged with primary anti‐Tom20 rabbit monoclonal antibodies (A19403, ABclonal, China) and anti‐α‐tubulin polyclonal antibodies (ab18251, Abcam, England), respectively. Subsequently, polyclonal AF647‐labeled goat anti‐rabbit IgGs (A21245, Invitrogen, USA) were applied as secondary antibodies. The labeling procedures were carried out as previously described [45].

### 4.8 Zebrafish Husbandry and Sample Preparation

The zebrafish facility and the work in this manuscript have been approved by the Animal Research Advisory Committee of Xidian University. Zebrafish were maintained according to the guidelines of the Institutional Animal Care and Use Committee. They were raised in a recirculating water system at 27 ± 1 °C, pH = 7.5, and exposed to a photoperiod of 16/8 h light/dark. 24 h post fertilization (hpf), embryos were incubated in egg water containing 0.045% 1‐phenyl‐2‐thiourea (PTU, P7629, Sigma‐Aldrich, USA) to prevent melanin pigment formation; egg water was exchanged every day.

At 72 hpf, zebrafish larvae were anesthetized in calcium‐free and magnesium‐free PBS (pH 7.4) containing 0.02% tricaine (E10521, Sigma‐Aldrich, USA) and 1% BSA. They were fixed in pre‐warmed fixation buffer (28 °C) for 10 min, prepared with the High‐fidelity Fixation Kit for cell actin (Sunbloss, HX‐FFKCA‐014, Standard Imaging, China). Afterward, the cells were permeabilized for 3 min in PBS with 0.1% Triton X‐100, stained with 0.3 µm Abberior STAR RED phalloidin (STRED‐0100‐20UG, Abberior, Germany) overnight, and stored at 4 °C in the dark prior to imaging (Figure 5).

Fertilized eggs of the transgenic zebrafish line *Tg(mnx1:mGFP)^ml3^* were obtained from the China Zebrafish Resource Center and maintained in E3 embryo medium supplemented with 0.003% PTU. For imaging (Figure 6), larvae at 72 hpf were anesthetized with 600 µm tricaine and embedded in 1% low melting‐point agarose (50080, Lonza, Switzerland).

### 4.9 Preparation of Mouse Tissue Slices

Transgenic mice (T006163, C57BL/6JGpt‐H11^em1Cin(^ *^CAG‐LoxP‐ZsGreen‐Stop‐LoxP‐tdTomato^* ^)^/Gpt) were purchased from GemPharmatech Co., Ltd. Transgenic mice (007788, STOCK Tg(Thy1‐EGFP)MJrs/J, The Jackson Laboratory, USA) were provided by Jiavis (Wuhan) Biomedical Co., Ltd. Tissue slices were prepared in the experimental animal center at Xidian University according to the guidelines of the Institutional Animal Care and Use Committee. Animals were sedated with isoflurane before being perfused first with PBS, followed by 4% paraformaldehyde fixative (4% PFA) (158127, Sigma‐Aldrich, USA). After perfusion, the animals were dissected, and kidney or brain tissues were collected and fixed overnight at 4 °C in 4% PFA. Subsequently, the samples were rinsed twice with PBS to remove residual fixative. Tissue slices were incubated in FDISCO refractive index homogenization solution [46] (JA11012, Jarvisbio, China) for 1 h to reduce the scattering of the sample. Then, the tissues were placed on a coverslip, infiltrated with 30 µL imaging solution [46] (JA3021, Jarvisbio, China), and covered with another coverslip on top. After sealing the edges of the samples with nail polish, the samples were ready for imaging.

## Author Contributions

S.A., X.G. and Z.C. performed all experiments and the data analysis. Y.L. designed and wrote the LabVIEW control program. X.K., K.W., H.S. contributed to data analysis. P.G. and G.U.N. conceived and supervised the project. S.A., X.G. and Z.C. wrote the first draft of the manuscript, P.G. and G.U.N. revised it, and the final version was written by G.U.N. with input from all authors.

## Conflicts of Interest

The authors declare no conflicts of interest.

## Supporting information




**Supporting File 1**: advs74516‐sup‐0001‐SuppMat.pdf.

**Supporting File 2**: advs74516‐sup‐0002‐Video S1.mp4.

**Supporting File 3**: advs74516‐sup‐0003‐Video S2.mp4.

**Supporting File 4**: advs74516‐sup‐0004‐Video S3.mp4.

**Supporting File 5**: advs74516‐sup‐0005‐Video S4.mp4.

**Supporting File 6**: advs74516‐sup‐0006‐Video S5.mp4.

## Data Availability

The data that support the findings of this study are available from the corresponding author upon reasonable request.

## References

[1] T. Araki , “The History of Optical Microscope,” Mechanical Engineering Reviews 4 (2017): 16–00242.

[2] E. Abbe , “Beiträge zur Theorie des Mikroskops und der Mikroskopischen Wahrnehmung,” Archiv für Mikroskopische Anatomie 9 (1873): 413–468.

[3] L. Schermelleh , A. Ferrand , T. Huser , et al., “Super‐resolution Microscopy Demystified,” Nature Cell Biology 21 (2019): 72–84. 30602772 10.1038/s41556-018-0251-8

[4] K. Nienhaus and G. U. Nienhaus , “Where Do We Stand With Super‐Resolution Optical Microscopy?,” Journal of Molecular Biology 428 (2016): 308–322. 26743847 10.1016/j.jmb.2015.12.020

[5] L. Gao , L. Shao , C. D. Higgins , et al., “Noninvasive Imaging Beyond the Diffraction Limit of 3D Dynamics in Thickly Fluorescent Specimens,” Cell 151 (2012): 1370–1385. 23217717 10.1016/j.cell.2012.10.008 PMC3615549

[6] S. W. Hell and J. Wichmann , “Breaking the Diffraction Resolution Limit by Stimulated Emission: Stimulated‐Emission‐Depletion Fluorescence Microscopy,” Optics Letters 19 (1994): 780–782. 19844443 10.1364/ol.19.000780

[7] U. V. Nägerl , K. I. Willig , B. Hein , S. W. Hell , and T. Bonhoeffer , “Live‐Cell Imaging of Dendritic Spines by STED Microscopy,” Proceedings of the National Academy of Sciences 105 (2008): 18982–18987. 10.1073/pnas.0810028105 PMC2585941 19028874

[8] A. von Diezmann , Y. Shechtman , and W. E. Moerner , “Three‐Dimensional Localization of Single Molecules for Super‐Resolution Imaging and Single‐Particle Tracking,” Chemical Reviews 117 (2017): 7244–7275. 28151646 10.1021/acs.chemrev.6b00629 PMC5471132

[9] M. Lelek , M. T. Gyparaki , G. Beliu , et al., “Single‐Molecule Localization Microscopy,” Nature Reviews Methods Primers 1 (2021): 39. 10.1038/s43586-021-00038-x PMC9160414 35663461

[10] R. Heintzmann and C. G. Cremer , “Laterally Modulated Excitation Microscopy: Improvement of Resolution by Using a Diffraction Grating,” Proc. SPIE 3568, Optical Biopsies and Microscopic Techniques III, (1999): 185–196.

[11] M. G. L. Gustafsson , “Extended Resolution Fluorescence Microscopy,” Current Opinion in Structural Biology 9 (1999): 627–628. 10508771 10.1016/s0959-440x(99)00016-0

[12] P. Byers , T. Kellerer , M. Li , Z. Chen , T. Huser , and T. Hellerer , “Super‐Resolution Upgrade for Deep Tissue Imaging Featuring Simple Implementation,” Nature Communications 16 (2025): 5386. 10.1038/s41467-025-60744-y PMC12198360 40562760

[13] R. Heintzmann and T. Huser , “Super‐Resolution Structured Illumination Microscopy,” Chemical Reviews 117 (2017): 13890–13908. 29125755 10.1021/acs.chemrev.7b00218

[14] X. Chen , S. Zhong , Y. Hou , et al., “Superresolution Structured Illumination Microscopy Reconstruction Algorithms: A Review,” Light: Science & Applications 12 (2023): 172. 10.1038/s41377-023-01204-4 PMC10336069 37433801

[15] M. G. L. Gustafsson , “Nonlinear Structured‐Illumination Microscopy: Wide‐Field Fluorescence Imaging With Theoretically Unlimited Resolution,” Proceedings of the National Academy of Sciences 102 (2005): 13081–13086. 10.1073/pnas.0406877102 PMC1201569 16141335

[16] R. Heintzmann , T. M. Jovin , and C. Cremer , “Saturated Patterned Excitation Microscopy—A Concept for Optical Resolution Improvement,” Journal of the Optical Society of America A 19 (2002): 1599–1609. 10.1364/josaa.19.001599 12152701

[17] O. Mandula , M. Kielhorn , K. Wicker , G. Krampert , I. Kleppe , and R. Heintzmann , “Line Scan—Structured Illumination Microscopy Super‐Resolution Imaging in Thick Fluorescent Samples,” Optics Express 20 (2012): 24167–24174. 23187180 10.1364/OE.20.024167

[18] B. E. Urban , J. Yi , S. Chen , et al., “Super‐Resolution Two‐Photon Microscopy via Scanning Patterned Illumination,” Physical Review E 91 (2015): 042703. 10.1103/PhysRevE.91.042703 PMC4565794 25974523

[19] B. E. Urban , L. Xiao , S. Chen , et al., “In Vivo Superresolution Imaging of Neuronal Structure in the Mouse Brain,” IEEE Transactions on Biomedical Engineering 65 (2018): 232–238. 29267161 10.1109/TBME.2017.2773540 PMC5967880

[20] E. Baumgart and U. Kubitscheck , “Scanned Light Sheet Microscopy With Confocal Slit Detection,” Optics Express 20 (2012): 21805–21814. 23037300 10.1364/OE.20.021805

[21] J. T. Frohn , H. F. Knapp , and A. Stemmer , “True Optical Resolution Beyond the Rayleigh Limit Achieved by Standing Wave Illumination,” Proceedings of the National Academy of Sciences 97 (2000): 7232–7236. 10.1073/pnas.130181797 PMC16528 10840057

[22] L. Wang , X. Zheng , J. Zhou , et al., “Improvement in Resolution of Multiphoton Scanning Structured Illumination Microscopy via Harmonics,” Engineering 16 (2022): 65–72.

[23] C. H. Yeh and S. Y. Chen , “Resolution Enhancement of Two‐Photon Microscopy via Intensity‐Modulated Laser Scanning Structured Illumination,” Applied Optics 54 (2015): 2309–2317. 25968516 10.1364/AO.54.002309

[24] C. H. Yeh , C. Z. Tan , C. H. A. Cheng , J. T. Hung , and S. Y. Chen , “Improving Resolution of Second Harmonic Generation Microscopy via Scanning Structured Illumination,” Biomedical Optics Express 9 (2018): 6081–6090. 31065414 10.1364/BOE.9.006081 PMC6490992

[25] G. Wen , S. Li , L. Wang , et al., “High‐Fidelity Structured Illumination Microscopy by Point‐Spread‐Function Engineering,” Light: Science & Applications 10 (2021): 12. 10.1038/s41377-021-00513-w PMC8016956 33795640

[26] M. Hüpfel , M. Fernández Merino , J. Bennemann , et al., “Two Plus One is Almost Three: A Fast Approximation for Multi‐View Deconvolution,” Biomedical Optics Express 13 (2022): 147–158. 35154860 10.1364/BOE.443660 PMC8803020

[27] A. Descloux , K. S. Grussmayer , and A. Radenovic , “Parameter‐Free Image Resolution Estimation Based on Decorrelation Analysis,” Nature Methods 16 (2019): 918–924. 31451766 10.1038/s41592-019-0515-7

[28] G. U. Nienhaus and J. S. Wiedenmann , “Dynamics and Optical Properties of Fluorescent Proteins: Perspectives for Marker Development,” Chemphyschem 10 (2009): 1369–1379. 19229892 10.1002/cphc.200800839

[29] J. Wiedenmann and G. U. Nienhaus , “Live‐Cell Imaging With EosFP and Other Photoactivatable Marker Proteins of the GFP Family,” Expert Review of Proteomics 3 (2006): 361–374. 16771707 10.1586/14789450.3.3.361

[30] H. Flanagan‐Steet , M. A. Fox , D. Meyer , and J. R. Sanes , “Neuromuscular Synapses Can Form In Vivo by Incorporation of Initially Aneural Postsynaptic Specializations,” Development 132 (2005): 4471–4481. 16162647 10.1242/dev.02044

[31] J. Li , X. Chen , K. Wen , et al., “Enhancing Optical Sectioning in Structured Illumination Microscopy With Axially Confined Fringe Modulation,” Laser & Photonics Reviews 19 (2025): 2401697.

[32] R. Dorn , S. Quabis , and G. Leuchs , “Sharper Focus for a Radially Polarized Light Beam,” Physical Review Letters 91 (2003): 233901. 14683185 10.1103/PhysRevLett.91.233901

[33] X. S. Xie , Y. Z. Chen , K. Yang , and J. Y. Zhou , “Harnessing the Point‐Spread Function for High‐Resolution Far‐Field Optical Microscopy,” Physical Review Letters 113 (2014): 263901. 25615335 10.1103/PhysRevLett.113.263901

[34] M. Hüpfel and G. U. Nienhaus , “Beam Shaping in Light‐Sheet Microscopy: An Experimental Analysis,” Journal of Physics: Photonics 6 (2024): 035003.

[35] J. Zheng , X. Fang , K. Wen , et al., “Large‐Field Lattice Structured Illumination Microscopy,” Optics Express 30 (2022): 27951–27966. 36236953 10.1364/OE.461615

[36] Y. Fu , Y. Hou , Q. Liang , et al., “Triangle‐Beam Interference Structured Illumination Microscopy,” Nature Photonics 19 (2025): 1122–1131.

[37] P. W. Winter , A. G. York , D. D. Nogare , et al., “Two‐Photon Instant Structured Illumination Microscopy Improves the Depth Penetration of Super‐Resolution Imaging in Thick Scattering Samples,” Optica 1 (2014): 181–191. 25485291 10.1364/OPTICA.1.000181 PMC4256096

[38] Y. Xue , J. R. Boivin , D. N. Wadduwage , J. K. Park , E. Nedivi , and P. T. C. So , “Multiline Orthogonal Scanning Temporal Focusing (mosTF) Microscopy for Scattering Reduction in in Vivo Brain Imaging,” Scientific Reports 14 (2024): 10954. 38740797 10.1038/s41598-024-57208-6 PMC11091065

[39] W. Ren , M. Guan , Q. Liang , et al., “Expanding Super‐Resolution Imaging Versatility in Organisms With Multi‐Confocal Image Scanning Microscopy,” National Science Review 11 (2024): nwae303. 40040644 10.1093/nsr/nwae303 PMC11879394

[40] K. Wicker and R. Heintzmann , “Resolving a Misconception About Structured Illumination,” Nature Photonics 8 (2014): 342–344.

[41] M. J. Booth , “Adaptive Optical Microscopy: The Ongoing Quest for a Perfect Image,” Light: Science & Applications 3 (2014): 165.

[42] N. Ji , “Adaptive Optical Fluorescence Microscopy,” Nature Methods 14 (2017): 374–380. 28362438 10.1038/nmeth.4218

[43] S. Dai , A. Kobitski , A. Barati Sedeh , S. Eroglu‐Kayikçi , L. Hilbert , and G. U. Nienhaus , “Photon‐Efficient Aberration Correction for 3D‐STED Imaging of Thick Biological Specimens Using Sensorless Adaptive Optics,” ACS Photonics 11 (2024): 310–320.

[44] F. Ströhl and C. F. Kaminski , “A Joint Richardson‐Lucy Deconvolution Algorithm for the Reconstruction of Multifocal Structured Illumination Microscopy Data,” Methods and Applications in Fluorescence 3 (2015): 014002. 29148478 10.1088/2050-6120/3/1/014002

[45] S. van de Linde , A. Löschberger , T. Klein , et al., “Direct Stochastic Optical Reconstruction Microscopy With Standard Fluorescent Probes,” Nature Protocols 6 (2011): 991–1009. 21720313 10.1038/nprot.2011.336

[46] Y. Qi , T. Yu , J. Xu , et al., “FDISCO: Advanced Solvent‐Based Clearing Method for Imaging Whole Organs,” Science Advances 5 (2019): aau8355. 10.1126/sciadv.aau8355 PMC6357753 30746463

