# Supplementary material for: Deep Volumetric Super‐Resolution Imaging in Thick Biological Specimens With Sparse Scanning SIM
Source: Adv Sci (Weinh). 2026 Feb 23;13(25):e74516. doi: 10.1002/advs.74516 (PMC13137830; doi:10.1002/advs.74516)
Supplement: Supplementary file 1 — Supporting File 1: advs74516‐sup‐0001‐SuppMat.pdf. [file ADVS-13-e74516-s004.pdf]

Supplementary Information

Deep volumetric super-resolution imaging in thick biological specimens with sparse scanning SIM

Sha An[1,2,3], Xuhong Guo[1,2,3], Zhongxia Cai[1,2,3], Yunze Lei[1,2,3], Kai Wen[1,2,3], Hongfei Suo[1,2,3], Xiaoyu Kong[1,2,3], G. Ulrich Nienhaus[4,5,6,7] *, Peng Gao[1,2,3, *]

[1] *School of Physics, Xidian University, Xi'an, 710071, China*
[2] *Key Laboratory of Optoelectronic Perception of Complex Environment, Ministry of Education, Xi'an, 710071, China*
[3] *Engineering Research Center of Information Nanomaterials, Universities of Shaanxi Province, Xi'an, 710071, China*
[4] *Institute of Applied Physics, Karlsruhe Institute of Technology, 76049 Karlsruhe, Germany*
[5] *Institute of Nanotechnology, Karlsruhe Institute of Technology, 76021 Eggenstein-Leopoldshafen, Germany*
[6] *Institute of Biological and Chemical Systems, Karlsruhe Institute of Technology, 76021 Eggenstein-Leopoldshafen, Germany*
[7] *Department of Physics, University of Illinois at Urbana-Champaign, Urbana, Illinois 61801, USA*
**uli@uiuc.edu (G. U. Nienhaus)*
**peng.gao@xidian.edu.cn (P. Gao)*

Contents

Supplementary Notes

Supplementary Note 1 | Synchronization of beam scanning with laser intensity modulation

Supplementary Note 2 | Fringe pattern simulation

Supplementary Note 3 | Characterization of sparse scanning structured illumination

Supplementary Note 4 | Fitting SS-SIM fringe patterns

Supplementary Note 5 | Harmonics and resolution enhancement of SS-SIM

Supplementary Note 6 | Simulation of conventional SIM and SS-SIM

Supplementary Note 7 | SS-SIM, WF and CLSM in 1PE imaging

Supplementary Note 8 | SS-SIM, WF and line-confocal imaging with 2PE

Supplementary Figures

Supplementary Figure 1 | Synchronization of beam scanning and laser power

Supplementary Figure 2 | Reticle imaging for measuring the coincidence of structured illumination and detection planes

Supplementary Figure 3 | Analysis of the relationship between fringe visibility and period

Supplementary Figure 4 | Optical sectioning strengths of conventional SIM and SS-SIM

Supplementary Figure 5 | FWHM analysis of sparse scanning fringes

Supplementary Figure 6 | Analysis of phase-shifted fringe patterns

Supplementary Figure 7 | Resolution analysis of 2PE SS-SIM

Supplementary Figure 8 | Effect of fringe period on the SBR of fluorescence images

Supplementary Figure 9 | Comparison of fringe patterns and frequency spectra of conventional SIM and SS-SIM

Supplementary Figure 10 | Fitting measured fringe patterns with Eq. (1) in the main text

Supplementary Figure 11 | Characterization of 1PE and 2PE SS-SIM harmonics

Supplementary Figure 12 | Comparison of the number of photons collected from a single emitter and the SBR of the reconstructed images for conventional SIM and SS-SIM

Supplementary Figure 13 | Schematic depiction of line confocal image reconstruction from SS-SIM raw images

Supplementary Figure 14 | Comparison of WF and SS-SIM images of bright emitters

Supplementary Figure 15 | Comparison of WF, SS-SIM and CLSM modalities

Supplementary Figure 16 | Comparison of 1PE CLSM and 2PE SS-SIM imaging

Supplementary Figure 17 | SS-SIM image reconstruction with the joint Richardson-Lucy deconvolution algorithm for multifocal structured illumination microscopy (jRL-MSIM)

Supplementary Videos

Supplementary Video 1 | 3D image stack of fluorescent microspheres embedded in agarose gel, taken with 1PE SS-SIM

Supplementary Video 2 | 3D image stack of the tail section of a fixed zebrafish

Supplementary Video 3 | 3D image stack of the eye of a fixed zebrafish

Supplementary Video 4 | 3D image stack of GFP-labeled zebrafish motoneurons

Supplementary Video 5 | 3D image stack of EGFP-labeled neurons in cleared mouse brain slices

Supplementary Tables

Supplementary Table 1 | Comparison of imaging properties and parameters of related scanning SIM-based modalities

Supplementary Notes

Supplementary Note 1 | Synchronization of beam scanning with laser intensity modulation

Sparse fringe patterns are generated by scanning a tightly focused laser beam with synchronized modulation of the laser intensity. With this scheme, arbitrary patterns can be written by the setup, including vertical and horizontal fringe patterns, as will be described in the following. For 2D beam steering, the DAQ issues voltages to the galvo mirror drivers, a sinusoidal signal to the RM and a staircase ramp to the GM for beam deflection in the x- and y-directions, respectively. Thus, the focus is steered in the x-direction according to

$$x(t) = A\cos(2\pi f t), \tag{1}$$

where t is the time, A is the scanning amplitude, $f = 12$ kHz is the resonant scanning frequency, so the period $T = f^{-1} = 83.33$ µs. After a complete RM cycle, the DAQ adds a voltage increment to the GM signal to advance a small step, Δy, along the y-direction, so that the y-position is given by

$$y(t) = \sum_{n=1}^{N} \Delta y \; H(t - n\,T), \tag{2}$$

with step counter, n, and Heaviside function, H. Disregarding the steps, Eq. (2) describes a linear ramp with slope $\Delta y/T$.

For synchronizing beam scanning with the laser intensity, a TTL signal is issued at the start of each RM scanning period as an external trigger for intensity modulation (**Supplementary Figure 1a**). For vertical fringe patterns, the DAQ board sends a pulse train to the laser, with pulse density inversely related to the scanning velocity of the

RM, so that spatially equidistant fringe patterns are generated along the y-direction (**Supplementary Figure 1b, d**). Notably, the fringe pattern along the x-direction is applied twice, once during forward and once during backward RM movement; the resulting images are superimposed. For horizontal fringe patterns, the laser is continuously on while the RM scans along the x-direction, and the GM steps synchronously along the y-direction, so that spatially equidistant fringe patterns are generated along the x-direction (**Supplementary Figure 1e**).

For recording a complete raw image, the DAQ generates a TTL signal to start and end sCMOS camera exposure at the rising and the falling edge, respectively. The camera is read out while the TTL signal is low. **Supplementary Figure 1c** shows the synchronization of camera recording, RM and GM scanning signals, and laser intensity modulation.

Supplementary Note 2 | Fringe pattern simulation

In SS-SIM, a larger fringe period yields a higher fringe visibility (contrast), since there is less crosstalk from neighboring fringes to the detected fluorescence intensities. However, more phase-shifted raw images are required to evenly cover the entire field of view (FOV). Thus, the chosen fringe period should balance imaging depth and speed. For fringe pattern optimization, we simulated these patterns as follows.

The 3D PSF intensity distribution was calculated with vectorial Debye theory[1],

$$E(r,\varphi,z)=\begin{bmatrix}E_x\\E_y\\E_z\end{bmatrix}=-\frac{ikf}{2\pi}\int_0^{\alpha}\int_0^{2\pi}A(\theta)\sin\theta\sqrt{\cos\theta}\times\exp\left[ik\left(z\cos\theta+r\sin\theta\cos(\phi-\varphi)\right)\right]$$
$$\times\begin{bmatrix}(\cos^2\phi\cos\theta+\sin^2\phi)\pm i\cos\phi\sin\phi(\cos\theta-1)\\ \cos\phi\sin\phi(\cos\theta-1)\pm i(\cos^2\phi+\sin^2\phi\cos\theta)\\ \sin\theta\exp(\pm i\phi)\end{bmatrix}\times d\phi d\theta \tag{3}$$

where r, φ, and z are the cylindrical coordinates of an observation point, ϕ is the azimuthal angle of the incident beam, $k=2\pi/\lambda$ is the wave vector (with wavelength $\lambda = \lambda_0/n$ for light with vacuum wavelength λ_0 propagating in a medium of refractive index n), f is the focal length of the high NA objective lens, and θ is the aperture angle varying between 0 and the maximum aperture angle, $\alpha = \arcsin(NA/n)$. The apodization function, $A(\theta)$, of the objective pupil was set to one in the calculation.

For the simulation, we took the experimental parameters, $NA = 1.2$, and $\lambda_0 = 0.64$ μm and 0.92 μm for 1PE and 2PE, respectively, and the refractive indices at these wavelengths were $n = 1.331$ and 1.322. The beam steering patterns with period p were convolved with the intensity of the 3D PSF for 1PE and its square for 2PE, and integrated along the axial direction, yielding the effective fringe intensity patterns observed on the sCMOS camera (**Fig. 1b**).

Supplementary Note 3 | Characterization of sparse scanning structured illumination

3.1 Coincidence of structured illumination and detection planes

To assess the coincidence of the planes of structured illumination and detection, we imaged a scale reticle using sparse fringe patterns. The opaque lines of the reticle and the scanned fringe patterns are simultaneously captured by the camera. The objective, MO (**Fig. 1**), was adjusted so as to obtain the sharpest image on the camera. Then, by iteratively fine-tuning the position of the scan lens, SL, along the axial direction, we minimized the full width at half maximum (FWHM) of the fringes to obtain best coincidence of structured illumination and detection planes. In this case, the sharpest fringes and the most defined reticle lines can be achieved, as shown in **Supplementary Figure 2**.

3.2 Relationship between the fringe visibility and period

Visibility (contrast) is a critical factor in the reconstruction of super-resolution images. Sparse fringe patterns result in a better visibility than dense (e.g., sinusoidal fringes) patterns used in traditional SIM, as more distant neighboring fringes contribute less background intensity. To systematically analyze the dependence of the visibility on the fringe period, we captured images of sparse fringe patterns with periods increasing by 0.2 μm in the range between 0.4 and 3.0 μm (**Supplementary Figure 3**). The images directly visualize that the contrast improves with increasing fringe period. For

quantification, we extracted intensity profiles perpendicular to the fringe orientations and analyzed the fringe visibility,

$$V = \frac{I_{max} - I_{min}}{I_{max} + I_{min}}. \tag{4}$$

Here, I_{max} and I_{min} are the (average) intensities of the peaks and the background, respectively. The fringe visibility is plotted versus the period in **Supplementary Figure 3e**. The visibility increases monotonically with period, and its slope decreases, as expected. It is evident that a period of 2.4 μm, which we chose in our SS-SIM implementation, captures most of the contrast enhancement feasible.

3.3 Optical sectioning capabilities of the conventional SIM and SS-SIM

In conventional SIM, fringe patterns are generated by two-beam interference, whereas SS-SIM uses focus scanning. For comparison, we experimentally assessed the optical sectioning performance of the two techniques. In general, optical sectioning is governed by the product of the illumination point spread function (PSF_{illum}) and the detection point spread function (PSF_{det}). As both methods have the same PSF_{det}, we only compare the axial extension of PSF_{illum}.

To ensure a fair comparison, we set the fringe period of conventional SIM to $p = 0.45$ μm, with which the resolution enhancement is 1.6, matching that of SS-SIM. Then, the spatial resolution enhancement is estimated as $\left(\frac{2NA}{\lambda} + \frac{1}{p}\right) / \frac{2NA}{\lambda} = \left(\frac{2\times1.2}{0.64} + \frac{1}{0.45}\right) / \frac{2\times1.2}{0.64}$, with $NA = 1.2$ and light wavelength, $\lambda = 0.64$ μm (Ref. 2).

Conventional SIM was performed on a DMD-based SIM microscope[3] with coherent illumination and a fringe period of 0.45 μm. The same water-immersion objective (HC

PL APO 63× NA 1.2) was used as for SS-SIM. A silver mirror, placed at the focal plane, was scanned in a range of 15 μm in axial steps of 200 nm. SS-SIM image acquisition and reconstruction were performed as describe in the main text. For conventional SIM, 3 (phase shifts) × 2 (orientation) raw images were recorded; reconstruction followed the procedure described previously[4].

The *xz*-sections of the fringe patterns of conventional SIM and SS-SIM are shown in **Supplementary Figure 4a, b**. After super-resolution reconstruction, the conventional SIM and SS-SIM images of the mirror surface are shown in **Supplementary Figure 4c, d**. Furthermore, axial intensity profiles along the dashed lines in **Supplementary Figures 4c** and **d** were extracted and fitted with Gaussians in **Supplementary Figures 4e** and **f**. Axial FWHMs of 5.65 ± 0.06 μm for conventional SIM and 0.67 ± 0.02 μm for SS-SIM were obtained from the fits, yielding an eightfold superior optical sectioning capability of SS-SIM.

3.4 FWHM analysis of scanned fringes

Resolution enhancement and optical sectioning crucially depend on the width of the bright fringes. To examine the sparse fringe patterns, a flat, silver-coated mirror was placed in the detection plane of the microscope. After adjusting the MO to yield the finest fringes on the camera, we measured the FWHM values of the *x*-oriented and *y*-oriented fringes (**Supplementary Figure 5a, b**) by fitting the intensity profiles perpendicular to the fringes with Gaussians (**Supplementary Figure 5c, d**), and the PSF of the SS-SIM system was determined by imaging fluorescent microspheres

(diameter 40 nm) under WF illumination (**Supplementary Figure 5e**). FWHM values each were determined at 15 randomly chosen positions for *x*-oriented fringes, *y*-oriented fringes, and the PSF, yielding averages and standard deviations (mean ± s.d.) of 0.293 ± 0.014 µm, 0.291 ± 0.015 µm and 0.349 ± 0.017 µm, respectively (**Supplementary Figure 5f**).

3.5 Phase-shifting of sparse illumination patterns

In SS-SIM, multiple raw images are acquired with precisely shifted fringe patterns to evenly expose the entire FOV. To examine these patterns in the horizontal and vertical orientations, we scanned the laser beam across a silver-coated mirror in the detection plane so as to produce two sets of 12 patterns displaced in steps of 200 nm (**Supplementary Figure 6a, b**); **Supplementary Figure 6c, d** displays intensity profiles perpendicular to the fringe orientations. Gaussian fits yielded an average lateral increment of 197 ± 13 nm (mean ± s.d.) for horizontal fringes and 198 ± 10 nm (mean ± s.d.) for vertical fringes, confirming precise phase shifting in both orientations.

3.6 Effect of fringe period on the SBR of raw fluorescence images

We analyzed the effect of the fringe period on the SBR of raw fluorescence images by imaging a sparse layer of fluorescent microspheres on a cover slip, using SS-SIM fringe periods ranging from 0.4 to 3.0 µm in 0.2-µm steps (**Supplementary Figure 8**). We fitted Gaussians to the line profiles through the peaks of 15 individual microspheres, and calculated the SBR as the ratio of the peak intensity to the average (background)

intensity between 2σ and 3σ (σ denotes the standard deviation) from the peak on either side of the Gaussian. The SBR, averaged over all microspheres, increases with increasing fringe period in a similar way as the visibility, and again suggests that a period of 2.4 μm is a good choice for our sparse fringe patterns.

Supplementary Note 4 | Fitting SS-SIM fringe patterns

To validate the expression describing sparse fringe patterns (Eq. (1) in the main text), a mirror was placed in the focal plane, and images were taken with the sCMOS camera using sparse fringe illumination (using 640-nm light). **Supplementary Figure 10a** shows a series of 12 images with phase shifts 0, 2π/12, 4π/12, …, 22π/12. Of note, these shifts correspond to exactly two pixels on the sCMOS camera, and shifting by integer pixel numbers yields a higher signal-to-noise ratio (SNR) in the image reconstruction. Intensity profiles (**Supplementary Figure 10b**) were fitted with $I_n(r) = \sum_{m=1}^{M} 2I_m\left[1 + \cos\left(2\pi m k_p r + m\varphi_n + \varphi_{0m}\right)\right]$, i.e., Eq. (1) in the main text. In our SS-SIM system, featuring a FWHM of the PSF of 0.35 μm for 1PE (0.32 μm for 2PE) and a fringe period of 2.4 μm, the number of harmonics (with 1PE) is limited to $M = 5$ (**Supplementary Note 5**). The global fit of all intensity profiles yields excellent agreement with the model function (**Supplementary Figure 10b**), and the fit parameters (in the figure caption) indicate the expected decrease of the amplitudes of the harmonics with order.

Supplementary Note 5 | Harmonics and resolution enhancement of SS-SIM

5.1 Harmonics

The number of harmonics included in the image reconstruction is a crucial parameter for resolution enhancement, so we examined it in detail by simulating SS-SIM imaging of fluorescent beads (**Supplementary Figure 11**). First, intensity distributions of sparse fringes, $I_n(x,y)$, with n indexing the 12 different phase-shifted patterns, were calculated according to Eq. (1) (in the main text) with 2.4-µm fringe period. Second, raw images of fluorescent particles were simulated by random deposition of point objects in a plane, multiplication with $I_n(x,y)$ and convolution with the PSF (computed as described in **Supplementary Note 2**).

Supplementary Figure 11a, b shows the simulated intensity distribution and its Fourier transform of one of the raw images. A cut through the Fourier transform along the fringe vector direction reveals multiple harmonics as discrete peaks (**Supplementary Figure 11c**), with peak amplitudes strongly decreasing with the order (**Supplementary Figure 11d**). An experimental raw image is shown in **Supplementary Figure 11e**. A cut through its Fourier transform yields similar results as the simulated data, i.e., harmonics above order five contribute negligibly (**Supplementary Figure 11g, h**). Therefore, we included harmonics up to order five in the image reconstruction.

5.2 Resolution enhancement

The diffraction-limited resolution is given by the reciprocal of the optical system's

cutoff frequency k_0, with $k_0 = 2NA/\lambda_{em}$ for 1PE and $k_0 = NA/(0.38\lambda_{ex})$ for 2PE[5, 6], where NA is the numerical aperture of the objective lens, λ_{em} is the peak wavelength of the fluorescence emission spectrum, and λ_{ex} is the wavelength of the 2PE laser.

Including up to the M-th harmonics in the spectrum synthesis for image reconstruction, the highest attainable frequency is $k_{max} = k_0 + Mk_p$, with $k_p = 1/p$ denoting the spatial frequency of the sparse fringe pattern. Dividing this equation by k_0 yields the resolution enhancement factor, $\frac{k_{max}}{k_0} = 1 + M\frac{k_p}{k_0}$. Notably, the number of harmonics with significant amplitude likewise depends (logarithmically) on the ratio k_p/k_0.

For 1PE SS-SIM, with imaging parameters $NA = 1.2$, $\lambda_{em} = 0.68$ µm, $p = 2.4$ µm, $M = 5$, $k_0 = 2NA/\lambda_{em} = 3.53$ µm^{-1} and $k_p = 1/p = 0.42$ µm^{-1}, the resolution enhancement factor is 1.59, in line with our experimental findings (**Fig. 2g-i**).

For 2PE SS-SIM, with parameters $NA = 1.2$ (except **Fig. 6a**), $\lambda_{ex} = 0.92$ µm, $p = 2.4$ µm, and $M = 5$ (**Supplementary Figure 11i-p**), $k_0 = NA/(0.38\lambda_{ex}) = 3.43$ µm^{-1}, $k_p = 1/p = 0.42$ µm^{-1}, we obtain a resolution enhancement factor of 1.61. **Supplementary Figure 7** shows 2PE WF, 2PE WF (with deconvolution) and 2PE SS-SIM images of fluorescent microspheres (40 nm diameter). The ratio of the FWHMs of 2PE WF (with deconvolution) and 2PE SS-SIM provides an experimentally determined resolution enhancement factor of 1.59, in line with our calculation. Of note, for the data in **Figs. 3a** and **6a**, we used a long working distance objective with $NA = 1.1$, $k_0 = 3.15$ µm^{-1} is slightly smaller, so the resolution enhancement factor is 1.67.

Supplementary Note 6 | Simulation of conventional SIM and SS-SIM

We simulated SS-SIM and conventional SIM images to compare their performance. Conventional SIM typically employs sinusoidal fringe patterns for illumination, whereas SS-SIM uses sparse fringe patterns, which can be modeled as convolutions of a Gaussian profile with a periodic Dirac comb function. Accordingly,

$$I_{illum}^{\mathrm{SIM}}(\boldsymbol{r}, n) = I_0[1 + m\cos(2\pi k_0 \boldsymbol{r} + \varphi_n)] \quad \text{(conventional SIM)}, \tag{5}$$

$$I_{illum}^{\mathrm{SS-SIM}}(\boldsymbol{r}, n) = I_0 \exp\left(-\frac{r^2}{2\sigma_{ex}^2}\right) \otimes \left[Ш_P\left(\boldsymbol{r} - \frac{n-1}{N} \cdot P\right)\right] \quad \text{(SS-SIM)}, \tag{6}$$

with amplitude, I_0, modulation depth, m, fringe-normal spatial coordinate, $\boldsymbol{r}$, spatial frequency of the fringe pattern, k_0, and phase shift, φ_n, where n indicates the phase shift index. The standard deviation of the Gaussian fringe profile for fluorescence excitation, $\sigma_{ex} = \frac{\lambda_{ex}}{2 \cdot NA \cdot 2.355}$, (note that the full width at half maximum is $2.355\ \sigma_{ex}$), $\otimes$ denotes the convolution operation, $Ш_P$ represents the Dirac comb function with period P, and N is the total number of phase-shifting steps.

A fluorescently labeled sample can be described by a 2D spatial distribution of emitters, $S(\boldsymbol{r})$. Here we have simulated discrete fluorescent beads at random locations within the FOV,

$$S(\boldsymbol{r}) = \sum_i \delta(\boldsymbol{r} - \boldsymbol{r}_i), \tag{7}$$

where $\boldsymbol{r}_i$ denotes the position (coordinates x, y) of the i-th bead. Then, the image recorded by the detector is given by

$$D(\boldsymbol{r}) = [S(\boldsymbol{r}) \cdot I_{illum}(\boldsymbol{r})] \otimes PSF(\boldsymbol{r}) + B(\boldsymbol{r}), \tag{8}$$

where $PSF(\boldsymbol{r}) = \exp\left(-\frac{r^2}{2\sigma_{em}^2}\right)$, with $\sigma_{em} = \frac{\lambda_{em}}{2 \cdot NA \cdot 2.355}$, and $B(\boldsymbol{r})$ represents the background term. Here, $I_{illum}(\boldsymbol{r})$ represents either $I_{illum}^{\mathrm{SIM}}(\boldsymbol{r}, n)$ or $I_{illum}^{\mathrm{SS-SIM}}(\boldsymbol{r}, n)$, depending on whether we calculate raw images for conventional SIM or SS-SIM.

The simulation was performed on a 512 × 512 grid with a pixel size of 50 nm, excitation wavelength, λ_{ex} = 637 nm, emission wavelength, λ_{em} = 680 nm, and objective NA = 1.2. For conventional SIM, sinusoidal fringes were simulated with modulation depth m = 0.1, k_0= 2.86 μm^{-1}, and phase shift, $\varphi_n = \frac{2\pi}{3}(n-1)$. For SS-SIM, the fringe pattern period P was set to 2.4 μm with N =12 phase-shift steps. To quantitatively validate the advantages of SS-SIM in terms of background suppression, raw SIM and SS-SIM images were simulated with the same level of additive noise, the average of which was chosen to be 10% of the peak intensity of the simulated beads (**Supplementary Figure 12a, b**).

For SIM image reconstruction, a standard frequency-domain synthesis method was employed[4]. Briefly, the spectral components of orders 0 and ±1 were decoupled, shifted to their original positions in Fourier space and superimposed. Finally, the synthesized spectrum was transformed back to the spatial domain via inverse Fourier transform to generate the reconstructed super-resolution image (**Supplementary Figure 12c**). For SS-SIM, the super-resolution image (**Supplementary Figure 12d**) was recovered using the reconstruction algorithm described in the Methods section.

For comparison of conventional SIM and SS-SIM in regard to noise reduction, we analyzed line profiles of the intensity emitted by an individual bead illuminated either with sinusoidal fringe patterns and three-step phase shifting (SIM) or sparse fringe

patterns and 12-step phase shifting (SS-SIM) (**Supplementary Figure 12e, f**). The total photon emission of the fluorescent bead was calculated by integrating all intensity profiles for the two imaging modalities. The analysis revealed that the total SS-SIM photon count was ~2.23 times that of conventional SIM, since the bead is repeatedly illuminated in SS-SIM and thus emits more photons. Notably, proper 2D integration of photon counts for each bead yields even 4.97-fold (2.23^2) the number of photons for SS-SIM in comparison to SIM. We further calculated the SBR of the reconstructed images for both modalities, yielding ~18 and ~80 for SIM and SS-SIM, respectively, and thus a 4.4-fold enhancement for SS-SIM (**Supplementary Figure 12g**). Of note, the improved background suppression of SS-SIM over SIM in this simulation originates simply from the fact that more raw images contribute to the total intensity of a single emitter.

Supplementary Note 7 | SS-SIM, WF and CLSM in 1PE imaging

For a comparison, we imaged two different samples with all three modalities, (1) surfaces sparsely decorated with immobilized 40 nm-diameter fluorescent microspheres, and (2) a zebrafish eye. Experimental results are given in **Supplementary Figure 15**. The WF images were obtained by superimposing all raw SS-SIM images, and the CLSM images were obtained with a standard confocal microscope, thus displaying a different FOV. Of note, the type of water immersion objective was identical for both systems.

For 2D imaging of fluorescent microspheres, CLSM takes ~14.7 s for scanning a FOV of 70 × 70 μm^2, sampled by 700 × 700 pixels, using a pixel dwell time of 30 μs. SS-SIM takes ~0.65 s for the same FOV and, therefore, is 23 times faster than CLSM. We fitted Gaussians to line profiles of 15 microspheres to extract FWHM values of 246 ± 12 nm (mean ± s.d.) for WF (with deconvolution), 154 ± 12 nm (mean ± s.d.) for SS-SIM and 255 ± 12 nm (mean ± s.d.) for CLSM, indicating a ~1.6-fold enhancement of the lateral resolution with SS-SIM over WF or CLSM.

For 3D imaging of a zebrafish eye, a volumetric FOV of 140 × 140 × 60 μm^3 was selected for CLSM and SS-SIM. The volume was axially sectioned into 300 layers in steps of 0.2 μm. For a single layer, CLSM takes ~58.8 s to image 1400 × 1400 pixels, while SS-SIM requires ~0.88 s, demonstrating a 67-fold acceleration of data acquisition for SS-SIM over CLSM. Moreover, SS-SIM yields a comparable penetration depth and SBR as CLSM yet higher spatial resolution. The lack of optical sectioning of the WF modality is apparent from the enormous background in the images.

Supplementary Note 8 | SS-SIM, WF and line-confocal imaging with 2PE

Under 2PE, fluorescence excitation occurs exclusively near the focal center due to the nonlinear nature of two-photon absorption, thus reducing photodestruction of the sample. Axial sectioning in 2PE imaging relies on the axial confinement of the fluorescence excitation, while 1PE CLSM relies on a pinhole for axial confinement of the detected fluorescence. Light scattering of the long-wavelength, near-infrared excitation light is much reduced. These beneficial properties have made 2PE laser scanning microscopy a key technique for deep imaging into (neuronal) tissue[6, 7].

With our 920-nm near-infrared pulsed laser, the width of the 2PE PSF (FWHM of 2PE WF bead images in **Supplementary Figure 7**) is 0.32 μm, slightly less than the one with 1PE (**Supplementary Figure 5f**). Combining sparse illumination with 2PE enables reconstruction of SS-SIM, WF, and line-confocal images from a single set of raw data. For the latter modality, 2PE is superior to 1PE due to its more constrained excitation region, resulting from the nonlinear absorption process. Data processing for line-confocal images is illustrated in **Supplementary Figure 13**. Only the intensities of the central rows of horizontal and vertical fringes were extracted from the corresponding 12 raw images, effectively implementing a virtual slit-shaped spatial filter. The final confocal image was generated by superimposing all 24 filtered images.

Supplementary Figures

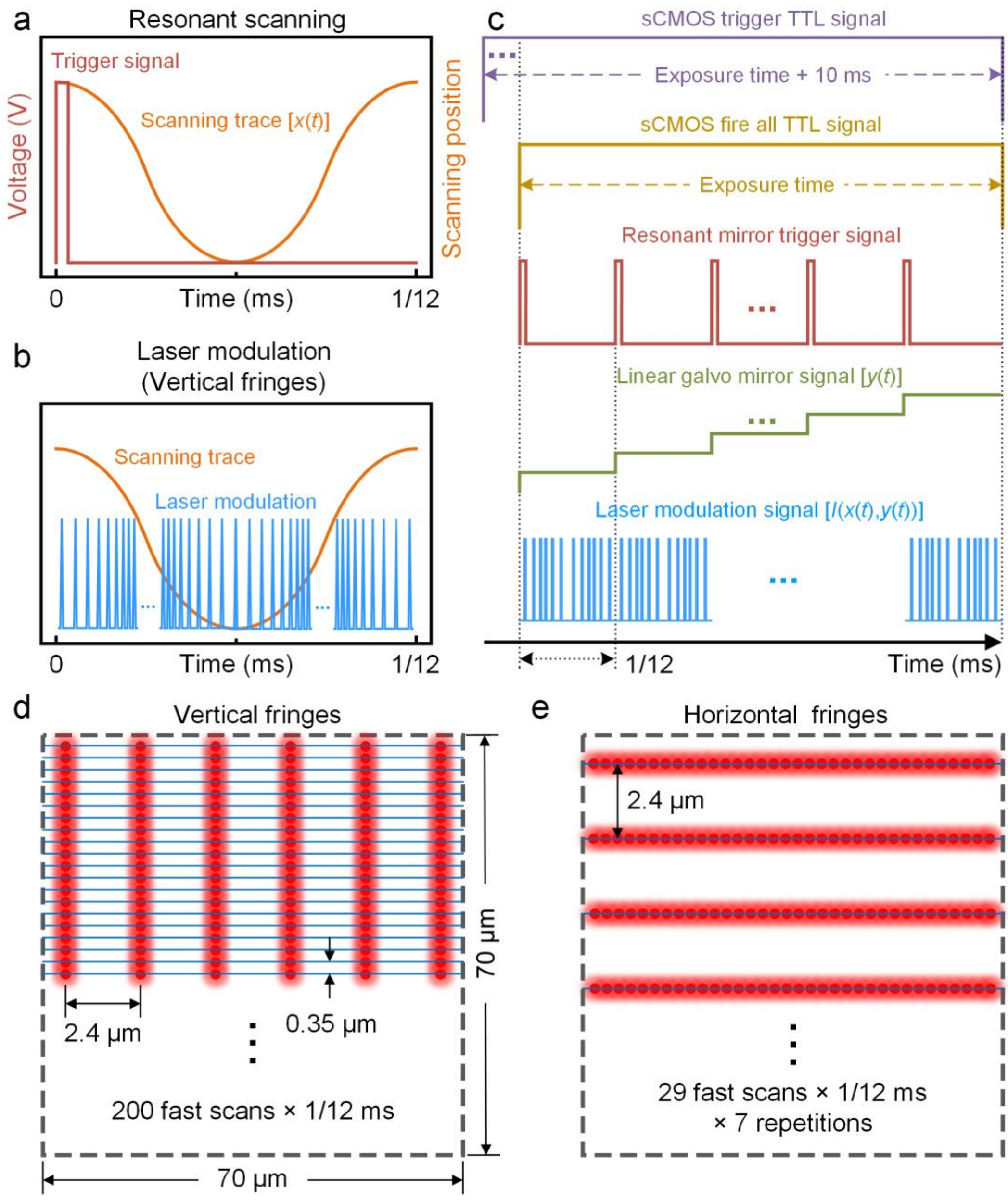

Supplementary Figure 1 | Synchronization of beam scanning and laser power. a Relationship between the resonant scanning mirror TTL synchronization signal and the scanning trace. **b** Relationship between the laser modulation signal for vertical fringes and the resonant scanning trace. **c** Diagram of the timing of the control signals for synchronization of beam scanning, laser modulation and camera readout. **d, e** Schematic of generating *y*-oriented (**d**) and *x*-oriented (**e**) fringes.

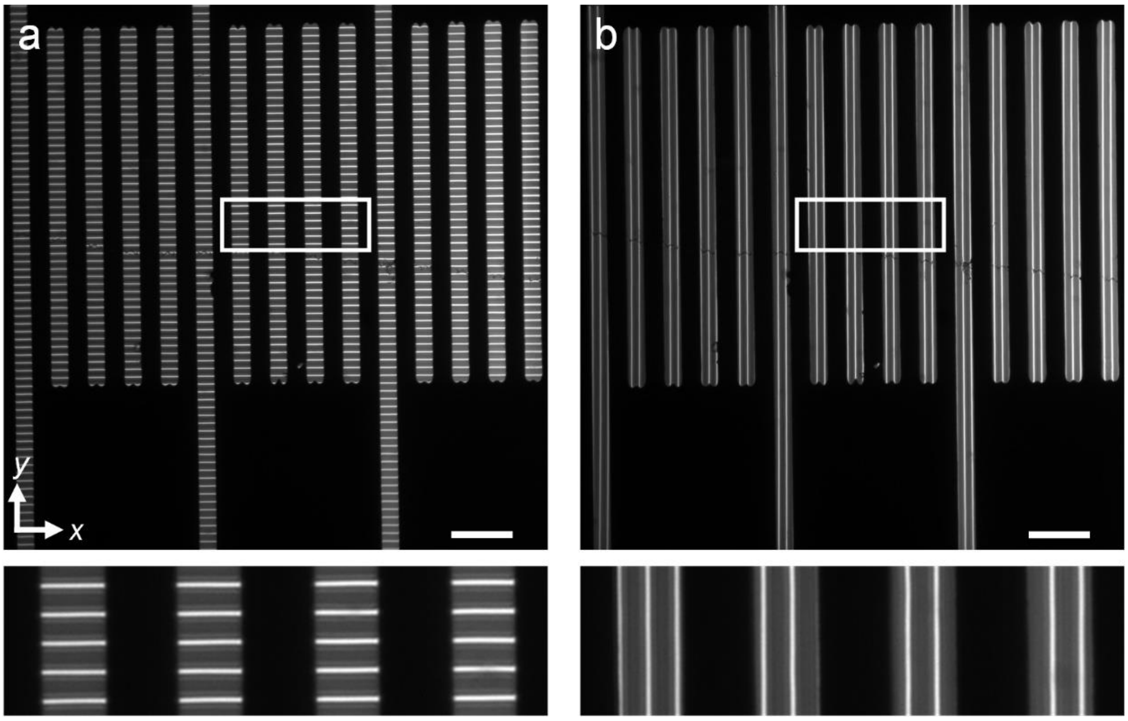

Supplementary Figure 2 | Reticle imaging for measuring the coincidence of structured illumination and detection planes. a, b Top: images of a reticle illuminated with horizontally (**a**) and vertically (**b**) oriented fringes, bottom: close-ups of the regions marked by rectangles. Scale bars, 15 µm.

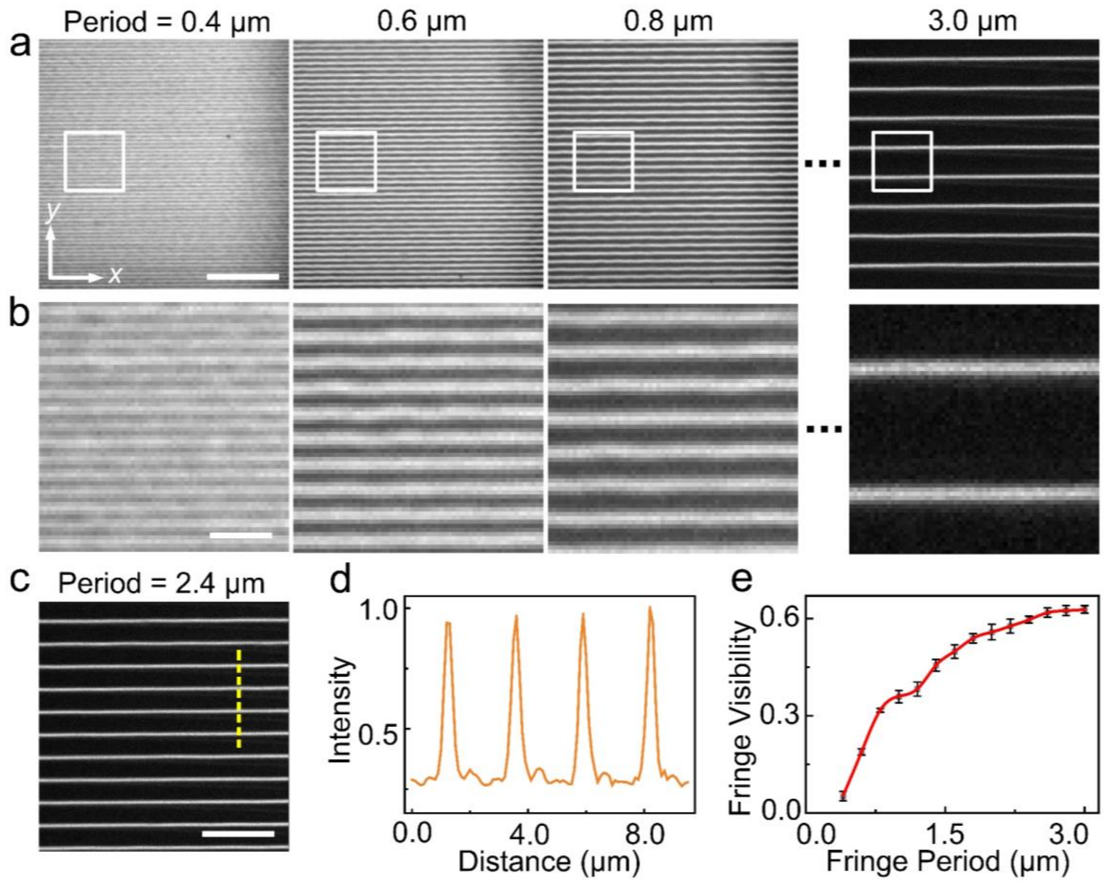

Supplementary Figure 3 | Analysis of the relationship between fringe visibility and period. a Horizontal fringe patterns with periods (shown on top) selected from a data set taken with periods between 0.4 and 3.0 μm in steps of 0.2 μm, measured by scanning a mirror positioned in the focal plane. Scale bar, 10 μm. **b** Enlarged views of the regions marked with rectangles in **a**. Scale bar, 2 μm. **c** Horizontal fringe pattern with a period of 2.4 μm. Scale bar, 10 μm. **d** Normalized intensity profile along the dashed line in panel **c**. **e** Fringe visibility, plotted as a function of the period; symbols, data (mean ± s.d. from 10 independent measurements); line, spline curve connecting the mean values.

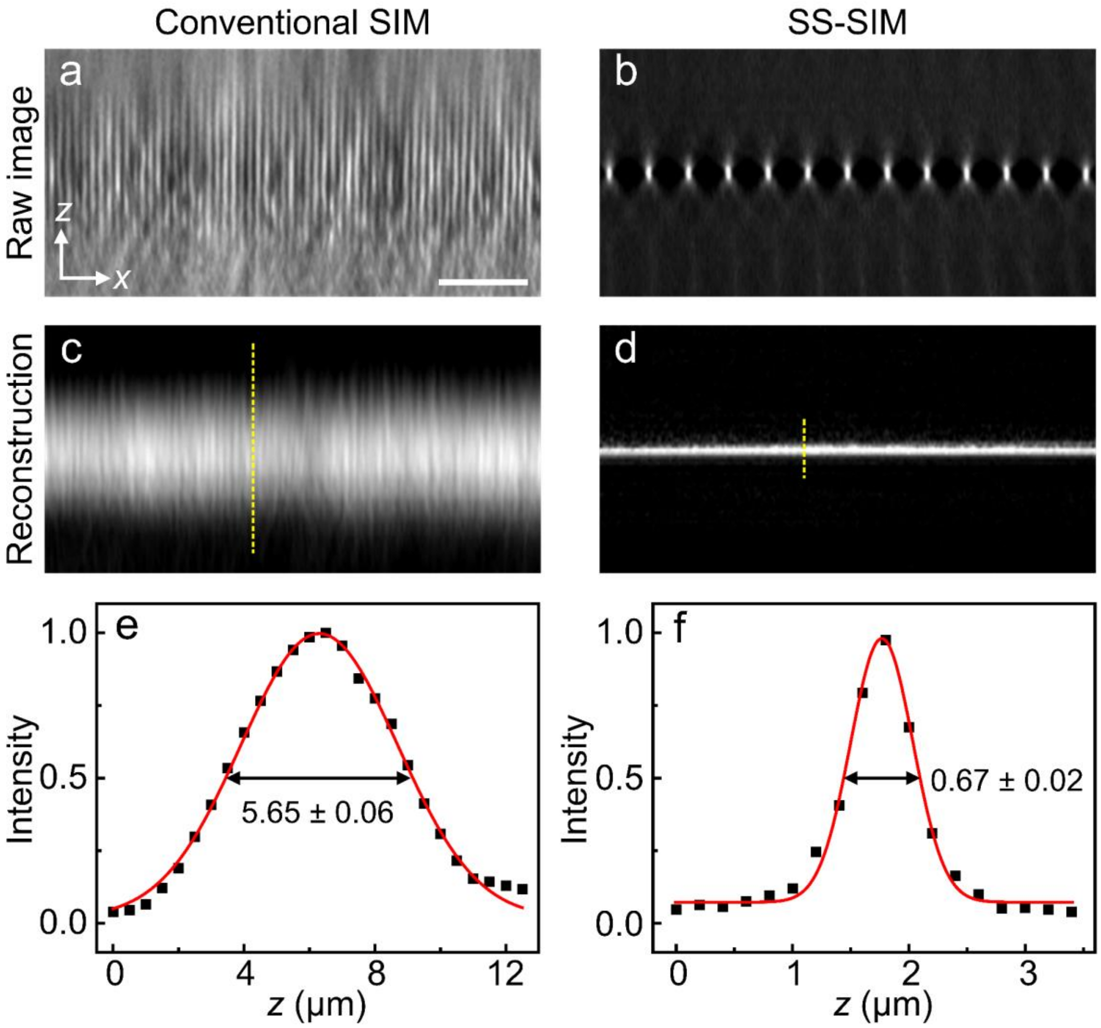

Supplementary Figure 4 | Optical sectioning strengths of conventional SIM and SS-SIM. a, b Fringe patterns (*xz*-sections) of conventional SIM (**a**) generated by two-beam interference, and of SS-SIM (**b**) generated by focus scanning, measured by 3D scanning a silver mirror surface; scale bar, 5 μm. **c, d** Image reconstructions by conventional SIM (**c**) and SS-SIM (**d**). **e, f** Normalized intensity profiles along the dashed lines marked in panels **c** and **d**, respectively; symbols, data; lines, Gaussian fits, yielding FWHM values as displayed in the panels.

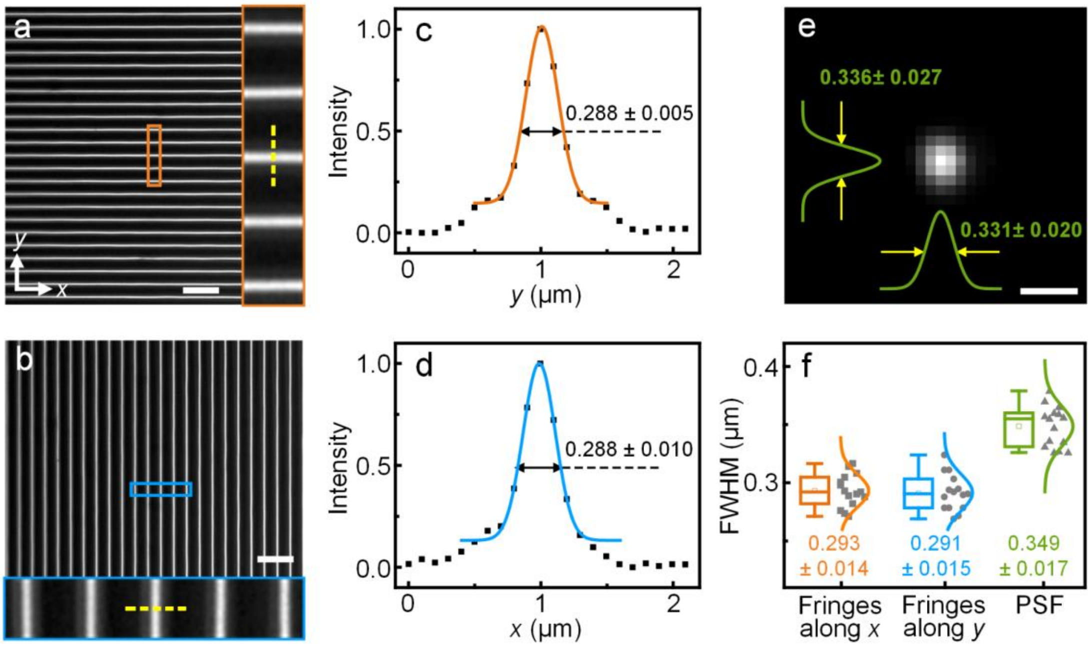

Supplementary Figure 5 | FWHM analysis of sparse scanning fringes. a, b Fringe patterns along the *x*-direction (**a**) and *y*-direction (**b**), including close-ups of the regions indicated by rectangles; scale bars, 5 µm. **c, d** Normalized intensity profiles along the dashed lines in **a** and **b**; symbols, data; lines, Gaussian fits, yielding FWHMs of the *x*-oriented and *y*-oriented fringes as shown in the panels. **e** Exemplary image of a 40-nm fluorescent microsphere under WF illumination. Line profiles of the particle through its center along the *x*- and *y*-axis were fitted with Gaussians, yielding FWHMs shown in the panel; scale bar, 0.5 µm. **f** FWHM values of *x*-oriented fringes (orange), *y*-oriented fringes (blue), and the system's PSF (green), with mean ± s.d. values (from 15 independent measurements) quoted in the figure.

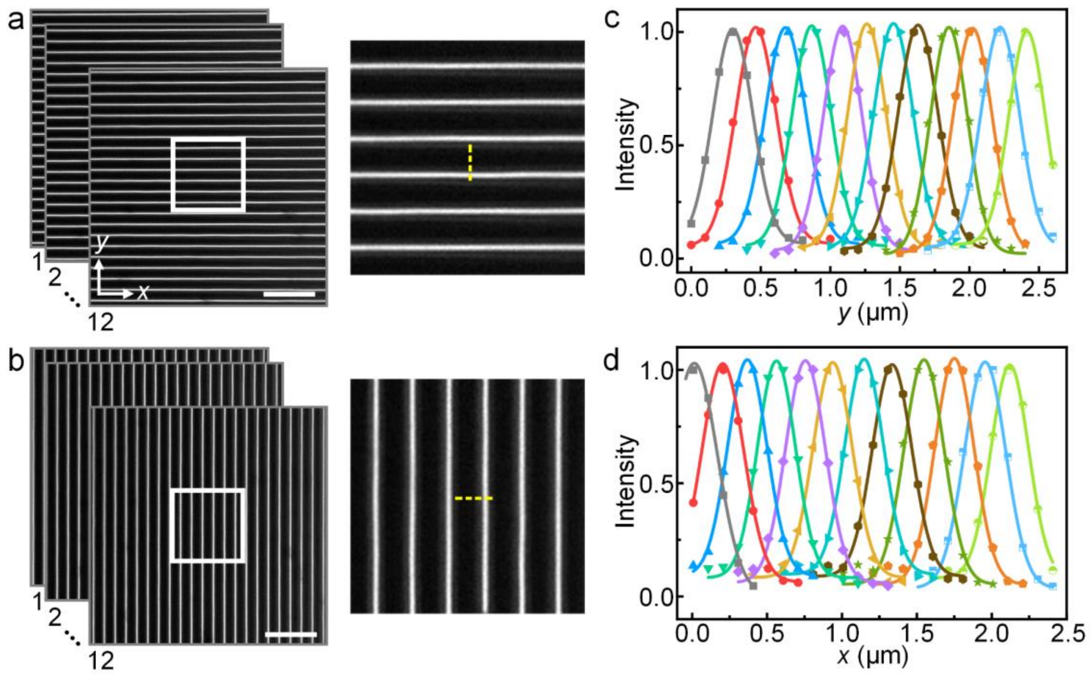

Supplementary Figure 6 | Analysis of phase-shifted fringe patterns. a, b Horizontal (**a**) and vertical (**b**) fringe patterns with 12 phase shift steps along the *y*-direction (**a**) and *x*-direction (**b**), with squares indicating close-up regions shown on the right. Scale bars, 10 μm. **c, d** Normalized intensity profiles (symbols) and Gaussian fit curves (lines) along the dashed lines in the close-ups of panels **a, b** for different phase shifts. The fringe patterns were recorded by scanning a silver-coated mirror in the focal plane.

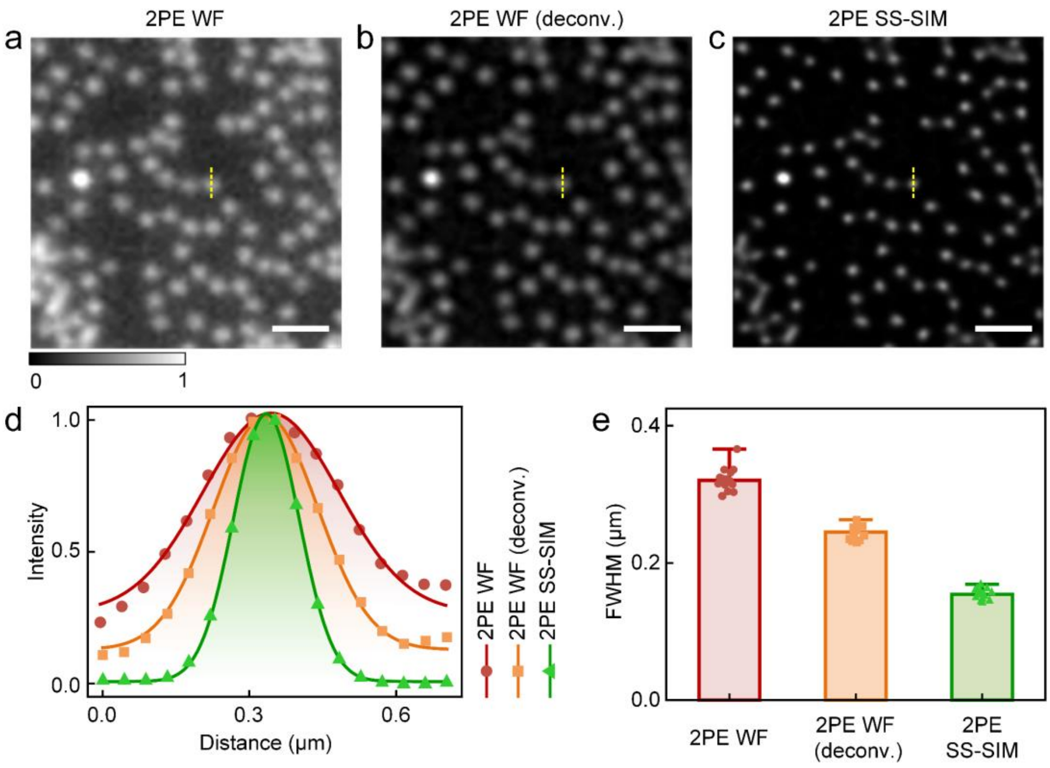

Supplementary Figure 7 | Resolution analysis of 2PE SS-SIM. a 2PE wide-field (WF), **b** the corresponding 2PE WF with deconvolution and **c** 2PE SS-SIM images of fluorescent microspheres (40 nm diameter); scale bars, 1 µm. **d** Normalized intensity profiles (symbols) along the dashed lines marked in panels **a**, **b** and **c**; Gaussian fits yield FWHM values of 326 ± 20 nm, 251 ± 6 nm and 159 ± 1 nm for 2PE WF, 2PE WF (with deconvolution), and 2PE SS-SIM, respectively. **e** FWHM values obtained by Gaussian fits to 15 randomly chosen intensity profiles, yielding 320 ± 17 nm (mean ± s.d.) for 2PE WF, 245 ± 10 nm (mean ± s.d.) for 2PE WF (with deconvolution), and 154 ± 7 nm (mean ± s.d.) for 2PE SS-SIM (with deconvolution). Symbols, individual microspheres, height of bars, mean; error bars, s.d..

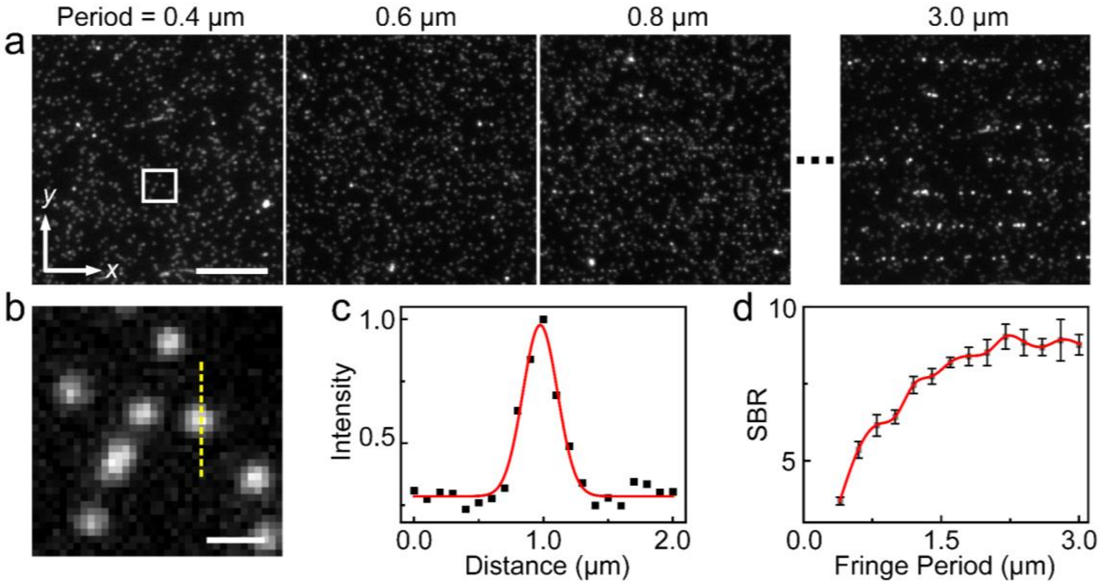

Supplementary Figure 8 | Effect of fringe period on the SBR of fluorescence images. a Raw images of 40 nm-diameter fluorescent microspheres with periods (shown on top) selected from a data set taken with periods between 0.4 and 3.0 μm in steps of 0.2 μm. Scale bar, 10 μm. **b** Enlarged view of the region marked by a square in **a**. Scale bar, 1 μm. **c** Normalized intensity profile (symbols) and Gaussian fit curve of an isolated microsphere along the dashed line marked in **b**. For calculating the SBR, the peak of the curve is taken as the signal, and the average of the 2σ – 3σ regions to the left and right of the curve is taken as the background. **d** Image SBR, plotted as a function of the fringe period; symbols, data (mean ± s.d. from 10 independent measurements); line, spline curve connecting the mean values.

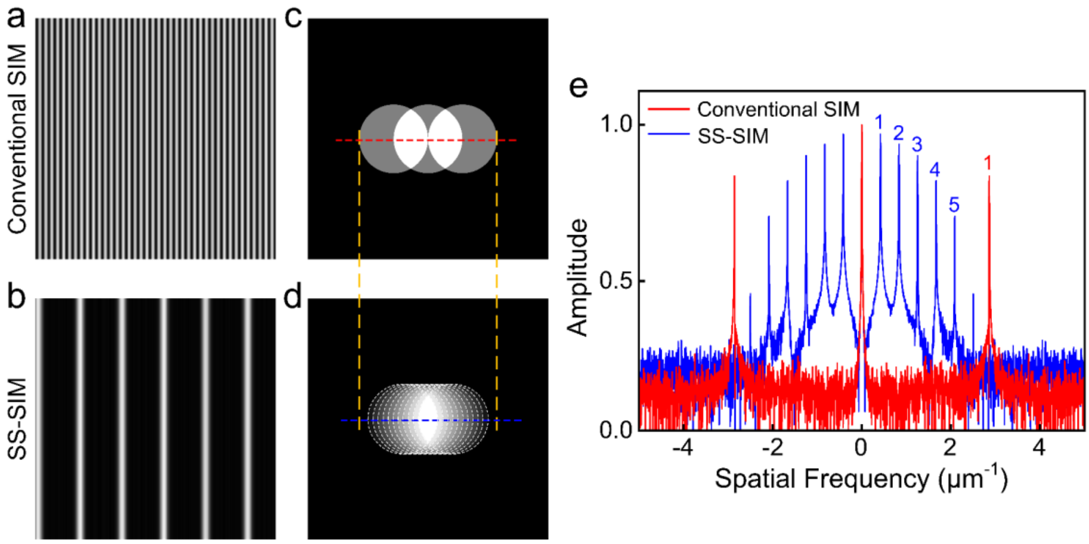

Supplementary Figure 9 | Comparison of fringe patterns and frequency spectra of conventional SIM and SS-SIM. a, **b** Fringe patterns and **c**, **d** spectra of SIM and SS-SIM, respectively. **e** Spectral amplitudes along the red and blue lines in panels **c** and **d**, respectively. The numbers above the peaks denote the diffraction orders.

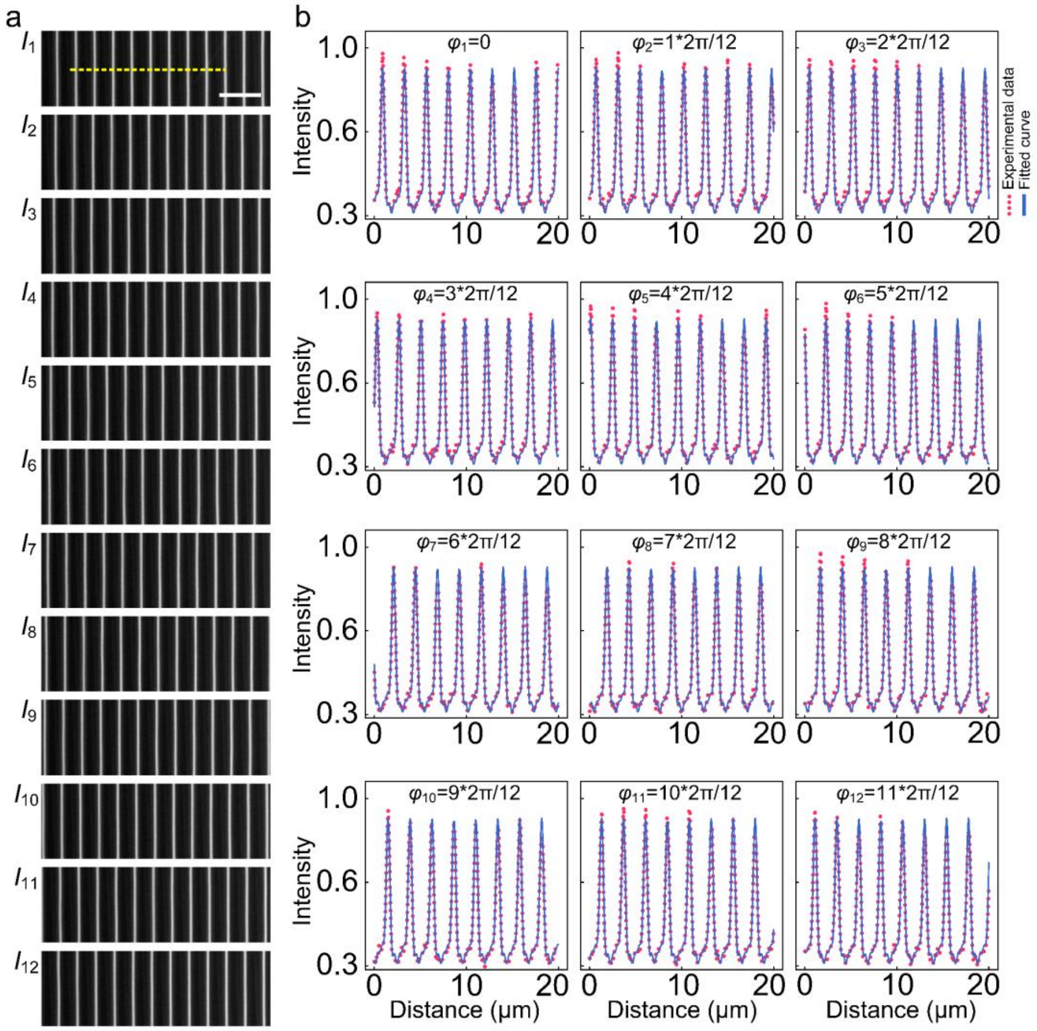

Supplementary Figure 10 | Fitting measured fringe patterns with Eq. (1) in the main text. a Phase-shifted fringe patterns obtained by scanning across a mirror in the sample plane. Scale bar, 5 μm. **b** Normalized intensity distributions along the dashed line in panel **a** (also applies to I_2 to I_{12}), symbols, data; lines, fits. A global fit of the first five intensity distributions with Eq. (1) in the main text yields $I_1 = 1488.21$, $I_2 = 918.16$, $I_3 = 522.61$, and $I_4 = 222.01$, $I_5 = 52.01$; $\varphi_{01} = 0.46$, $\varphi_{02} = 0.66$, $\varphi_{03} = 1.06$, $\varphi_{04} = 1.19$ and $\varphi_{05} = 1.16$ rad.

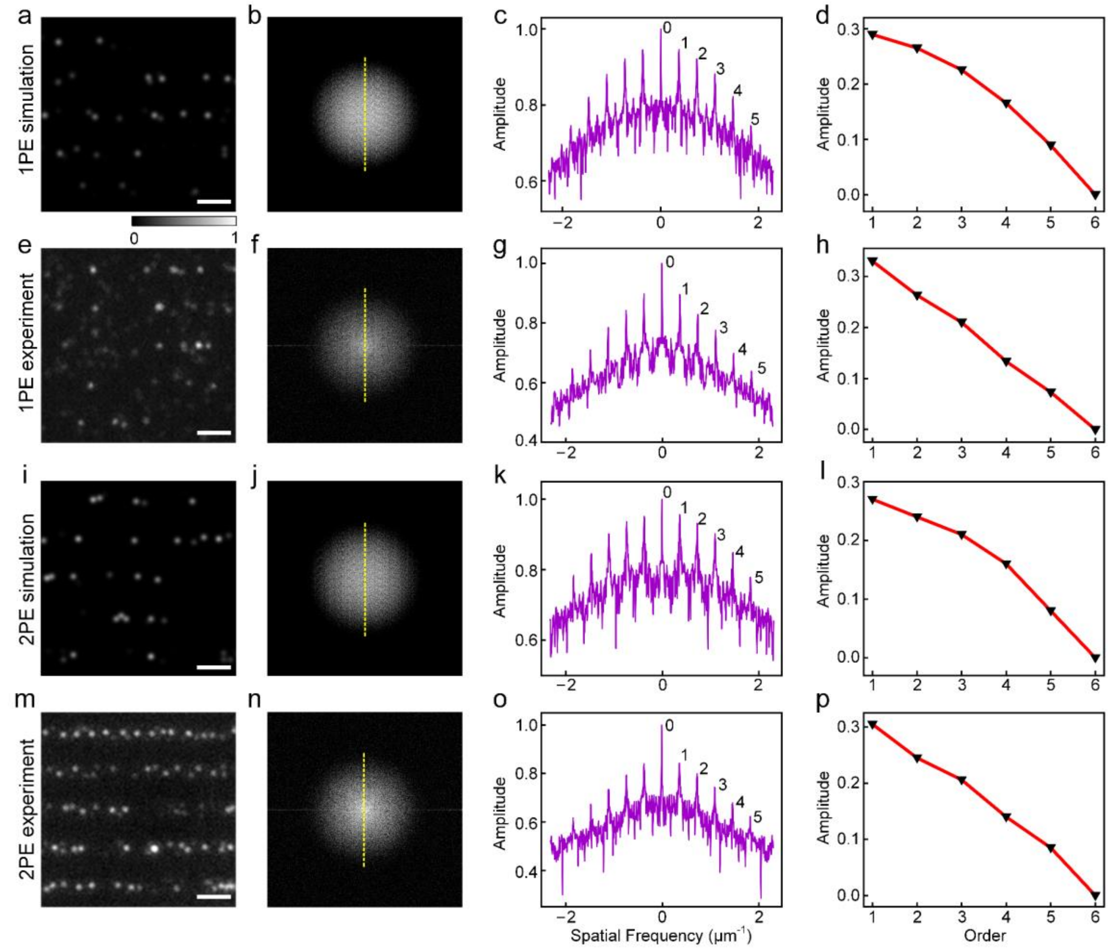

Supplementary Figure 11 | Characterization of 1PE and 2PE SS-SIM harmonics. The analysis is based on images of fluorescent microspheres, simulated/measured under sparse fringe illumination with 2.4-μm period**. a-d** Simulated and **e-h** experimental image data under 1PE. **a, e** Raw images (scale bar: 2 μm); **b, f** the corresponding Fourier transforms; **c, g** normalized amplitude profiles along the dashed lines in panels **b, f**, with peaks numbered according to the order of the harmonics; **d, h** background-corrected peak amplitudes, plotted as a function of the order. **i-l** Simulated and **m-p** experimental image data under 2PE. **i, m** Raw images (scale bar: 2 μm); **j, n** the corresponding Fourier transforms; **k, o** normalized amplitude profiles along the dashed lines in panels **j, n**, with peaks numbered according to the order of the harmonics; **l, p** background-corrected peak amplitudes, plotted as a function of the order.

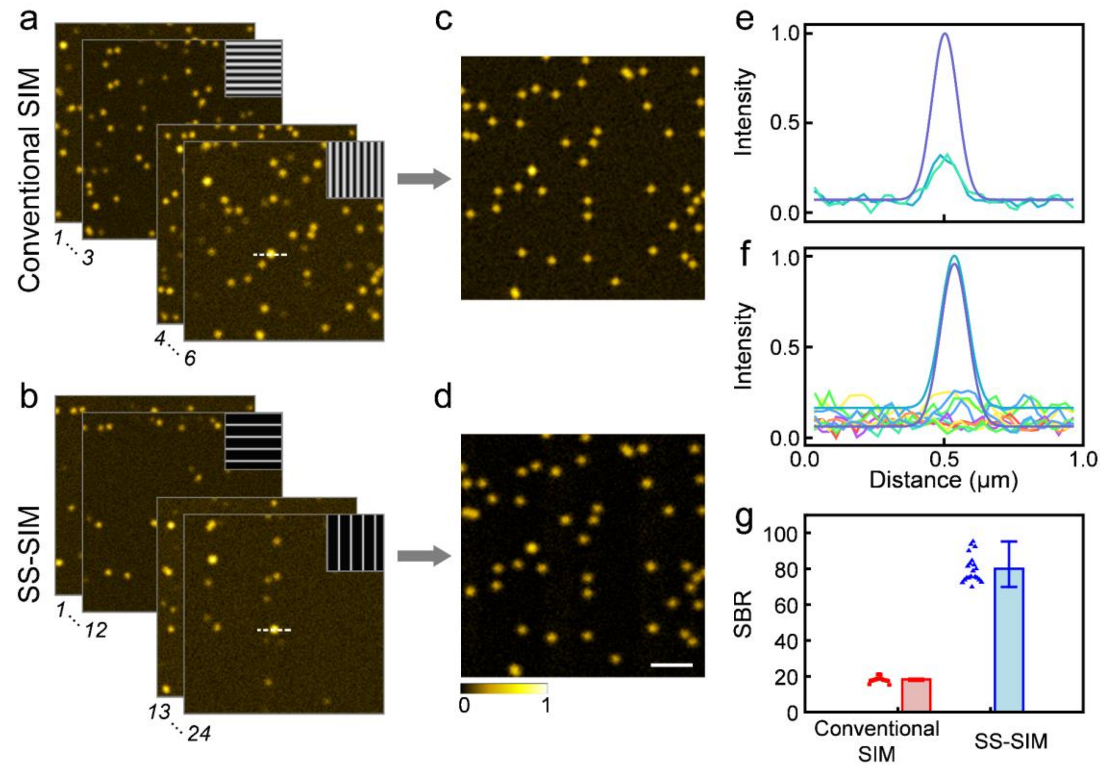

Supplementary Figure 12 | Comparison of the number of photons collected from a single emitter and the SBR of the reconstructed images for conventional SIM and SS-SIM. a, b Representative simulated raw SIM (**a**) and SS-SIM (**b**) images with the same noise levels. **c, d** Reconstructed SIM (**c**) and SS-SIM (**d**) super-resolution images. Scale bar, 1 µm. **e, f** Intensity profiles of a selected bead under SIM illumination with 3 phase steps (**e**) and SS-SIM illumination with 12 phase steps (**f**). The total photon count of SS-SIM is 2.23-fold larger than that of SIM, as obtained by integration of the intensity curves. **g** SBR statistics on 20 randomly selected fluorescent beads, yielding an average SBR of ~18 for conventional SIM and ~80 for SS-SIM. Symbols, individual beads; height of bars, mean; error bars, s.d..

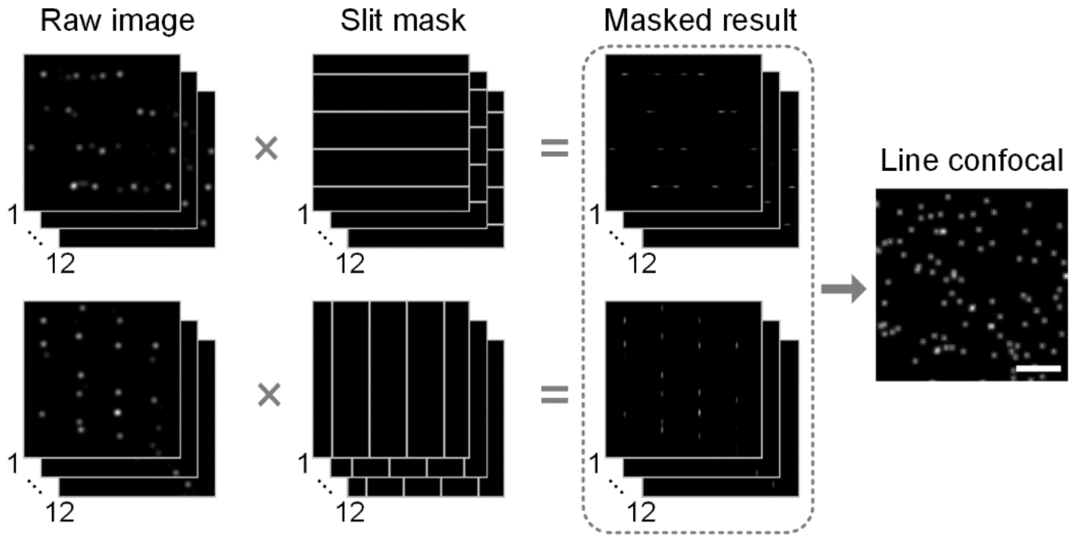

Supplementary Figure 13 | Schematic depiction of line confocal image reconstruction from SS-SIM raw images. Scale bar, 2 μm.

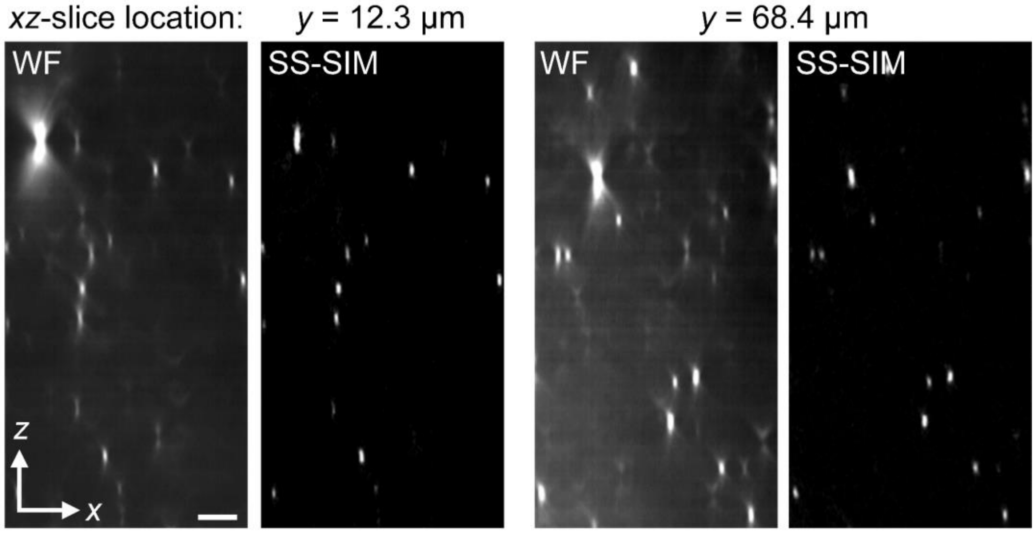

Supplementary Figure 14 | Comparison of WF and SS-SIM images of bright emitters. In this example, a bright cluster of fluorescent beads in a 3D phantom sample with fluorescent microspheres (40 nm diameter) immobilized in an agarose gel shows a large spread in the axial direction in the WF modality, unlike SS-SIM. Scale bar: 10 µm.

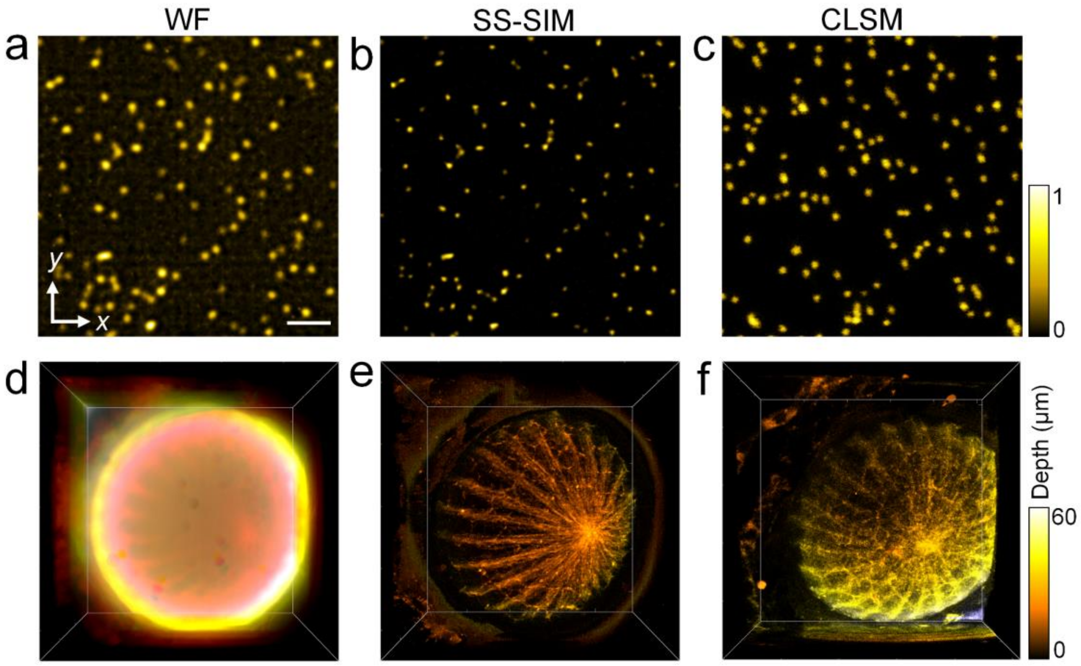

Supplementary Figure 15 | Comparison of WF, SS-SIM and CLSM modalities. a-c WF (**a**), SS-SIM (**b**) and CLSM (**c**) 2D images of a glass surface sparsely decorated with fluorescent microspheres of 40-nm diameter; scale bar, 2 μm; color bar, intensity encoding. **d-f** WF (**d**), SS-SIM (**e**) and CLSM (**f**) 3D image stacks (140 × 140 × 60 μm^3) of a zebrafish eye; color bar, depth encoding.

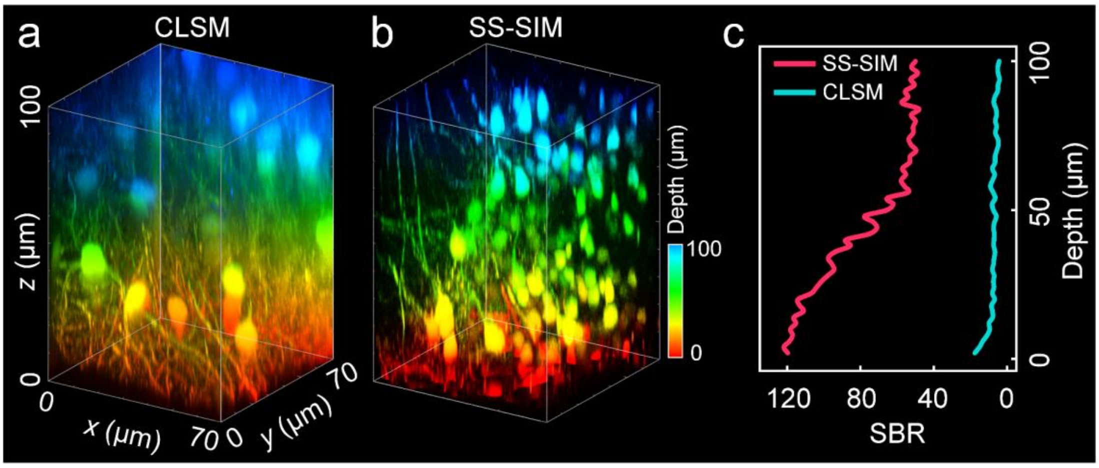

Supplementary Figure 16 | Comparison of 1PE CLSM and 2PE SS-SIM imaging. Volumetric images (70 × 70 × 100 μm^3) from a mouse brain, with neurons labeled via Thy-EGFP fusion proteins, taken with **a** 1PE CLSM and **b** 2PE SS-SIM; color bar, depth encoding. **c** SBR versus imaging depth for CLSM and SS-SIM.

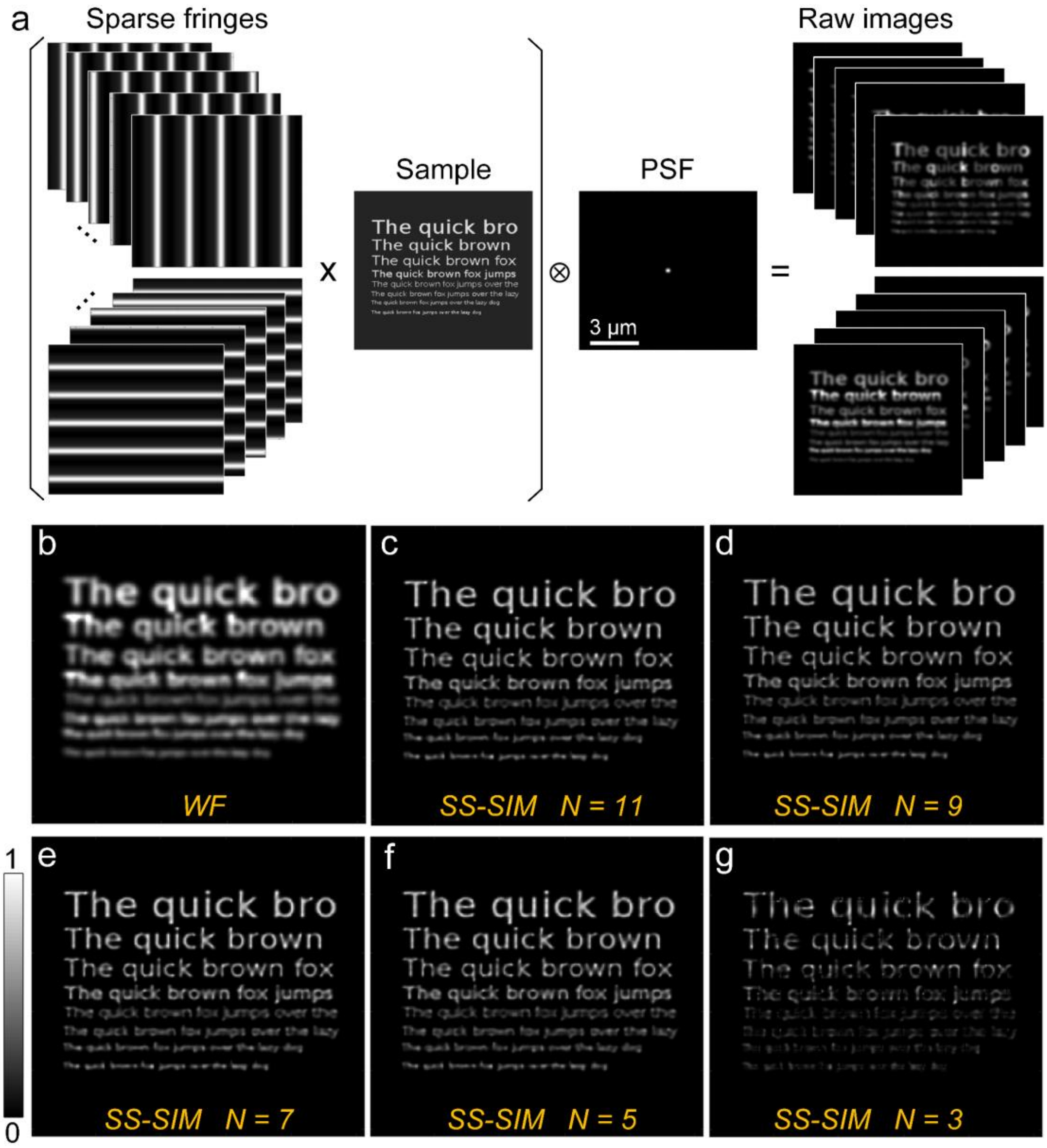

Supplementary Figure 17 | SS-SIM image reconstruction with the joint Richardson-Lucy deconvolution algorithm for multifocal structured illumination microscopy (jRL-MSIM). a Schematic of SS-SIM image simulation. Imaging parameters: The FWHM of the SS-SIM system PSF was set to 283 nm (at λ_{em} = 680 nm) and the fringe period to 2.4 µm. The image intensity distribution was modeled with Eq. (1); parameters are specified in **Supplementary Figure 10**. **b** Simulated WF image and **c-g** SS-SIM images reconstructed by the joint Richardson-Lucy algorithm, with numbers of raw images specified in the panels. In the calculation, the illumination function in the original jRL-MSIM algorithm[8], i.e., the delta comb function, was replaced with a sum of five cosine terms, as described in **Supplementary Figure 10**.

Supplementary Tables

Supplementary Table 1 | Comparison of imaging properties and parameters of related scanning SIM-based modalities

Technique	*Illumination*	*Resolution* / *Improvement over WF*	*Imaging Depth*	*Imaging Speed*	*Phototoxicity*
**Line-scan SIM**[9]	Single-line scanning, SIM grating	**~200 nm** / ~1.6-fold	**~5 µm** (63×/1.4 NA)	**Extremely slow** (~75 s/slice)	**Moderate** (raw data frames: 480 frames)
**2P ISIM**[10]	Single focus scanning	**~150 nm** / ~2-fold	**~110 µm** (60×/1.2 NA)	**Moderate** (~3 s/slice)	**High** (same as 2P laser scanning microscopy)
**mosTF**[11]	Multi-line scanning	**~850 nm** / No improvement	**~170 µm** (20×/1.0 NA)	**Moderate** (~4.1 s/slice)	**Moderate** (raw data frames: 256)
**MC-ISM**[12]	Multi-focus scanning	**110–130 nm** / ~2-fold	**~175 µm** (20×/0.7 NA)	**Very fast** (~0.06 s/slice)	**Extremely Low** (raw data frames: 25)
**SS-SIM**	Single focus scanning, sparse fringe patterns	**~150 nm** / ~1.6-fold	**~600 µm** (63×/1.2 NA and 40×/1.1 NA)	**Fast** (0.65 s/slice)	**Low** (raw data frames: 24)

References

1. Chen, B., Zhang, Z.M. & Pu, J.X. Tight focusing of partially coherent and circularly polarized vortex beams. *Journal of the Optical Society of America a-Optics Image Science and Vision* **26**, 862-869 (2009).
2. Brown, P.T., Kruithoff, R., Seedorf, G.J. & Shepherd, D.P. Multicolor structured illumination microscopy and quantitative control of polychromatic light with a digital micromirror device. *Biomedical Optics Express* **12**, 3700-3716 (2021).
3. Li, J. et al. Enhancing Optical Sectioning in Structured Illumination Microscopy With Axially Confined Fringe Modulation. *Laser & Photonics Reviews* **19**, 2401697 (2025).
4. Wen, K. et al. Large-field structured illumination microscopy based on 2D grating and a spatial light modulator. *Optics Letters* **47**, 2666-2669 (2022).
5. Bumstead, J.R. et al. Designing a large field-of-view two-photon microscope using optical invariant analysis. *Neurophotonics* **5** (2018).
6. Zipfel, W.R., Williams, R.M. & Webb, W.W. Nonlinear magic: multiphoton microscopy in the biosciences. *Nature Biotechnology* **21**, 1368-1376 (2003).
7. Wu, J.L. et al. Kilohertz two-photon fluorescence microscopy imaging of neural activity in vivo. *Nature Methods* **17**, 287-290 (2020).
8. Ströhl, F. & Kaminski, C.F. A joint Richardson-Lucy deconvolution algorithm for the reconstruction of multifocal structured illumination microscopy data. *Methods and Applications in Fluorescence* **3**, 014002 (2015).
9. Mandula, O. et al. Line scan - structured illumination microscopy super-resolution imaging in thick fluorescent samples. *Optics Express* **20**, 24167-24174 (2012).
10. Winter, P.W. et al. Two-photon instant structured illumination microscopy improves the depth penetration of super-resolution imaging in thick scattering samples. *Optica* **1**, 181-191 (2014).
11. Xue, Y. et al. Multiline orthogonal scanning temporal focusing (mosTF) microscopy for scattering reduction in in vivo brain imaging. *Scientific Reports* **14**, 10954 (2024).
12. Ren, W. et al. Expanding super-resolution imaging versatility in organisms with multi-confocal image scanning microscopy. *National Science Review* **11**, nwae303 (2024).
